# Comparison of the Structure of Advanced Organic Electrode Materials, TAPT and “TAQ”, by Multinuclear NMR and Stability Analysis

**DOI:** 10.1021/acs.jpca.6c03213

**Published:** 2026-07-17

**Authors:** J. M. Litterio, S. H. Finkelstein, Z. Sun, K. M. Cerri, K. Schmidt-Rohr

**Affiliations:** Department of Chemistry, 8244 Brandeis University, Waltham, Massachusetts 02453, United States

## Abstract

The chemical structure of two insoluble tricyclic organic molecules made from the same precursor through different reported synthesis routes and forming promising electrode materials is compared by advanced solid-state ^13^C, ^15^N, ^13^C­{^1^H}, ^15^N­{^1^H}, and ^15^N–^15^N nuclear magnetic resonance (NMR). One synthesis resulted in TAPT, tetraamino-phenazine-1,4,6,9-tetrone, while several prominent publications identified the product of the other procedure as TAQ, with fewer lithium binding sites due to hydrogen atoms bonded to two nitrogen atoms, which also make the central ring antiaromatic. Peak positions in the ^13^C and ^15^N NMR spectra of TAPT and two “TAQ” materials, including ^15^N_6_-“TAQ”, are similar and agree with chemical-shift predictions for TAPT. Differences in line broadening arise from different concentrations of unpaired electrons, confirmed by pronounced differences in spin–lattice relaxation times. Slow ^15^N­{^1^H} and long-range ^13^C­{^1^H} dipolar dephasing proves that like TAPT, “TAQ” does not contain central N–H groups. The aromatic pyrazine ring in TAPT and “TAQ” is confirmed by the 309 ppm chemical shift of the nonprotonated nitrogen atom. Moderate splittings of the ^15^NH_2_ and ^13^C–NH_2_ NMR signals are shown to be due to symmetry-lowering differences in hydrogen bonding. Mass spectra of TAPT and “TAQ” exhibit matching peaks. Quantum-chemical analyses show that formation of TAQ with its antiaromatic central ring is not thermodynamically favorable and that it would spontaneously tautomerize into a reduced form of TAPT. In conclusion, both TAPT and “TAQ” are tetraamino-phenazine-1,4,6,9-tetrone, and published studies of the properties of “TAQ” are therefore duplicative.

## Graphical abstract

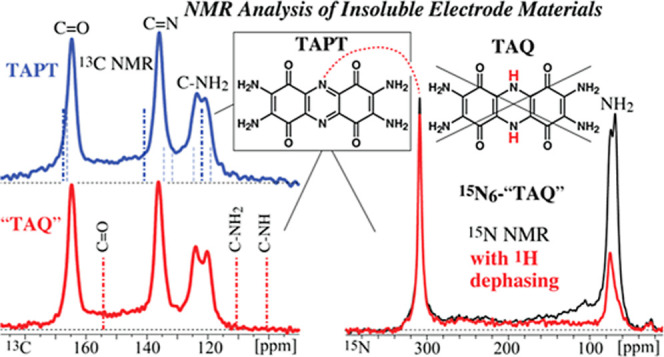

## Introduction

Lithium-ion batteries are notable for their rechargeability, high energy per mass relative to other battery types, a relatively long cycle life, moderate to good thermal stability, and good power capability.^1,2^ These properties can be tuned by choosing different materials for the positive electrode.^1,2^ Lithium nickel manganese cobalt oxide (NMC) and nickel cobalt aluminum oxide (NCA) have been dominant in the electric vehicle industry while lithium cobalt oxide (LCO) is common in portable phones, all with impressive energy and power density, cycle life, and stability,^1^ but labor-intensive and expensive to produce. Promising alternatives, such as organic electrode materials (OEMs) that can strongly bind lithium,^3^ have been the subject of extensive research over the past decade.^4–32^ Quinone- and nitrogen-rich molecules, in particular those with pyrazine cores, have attracted particular interest.^4–7^ Favorable properties of such molecules include strong hydrogen bonding and π–π stacking for insolubility in common battery solvents with dissolved LiPF_6_ ^8^ as well as several redox sites to strongly bind lithium, aided by aromatic stabilization of the lithium-bound forms. Among these materials are TAPT,^9–13,18^ 2,3,7,8-tetraaminophenazine-1,4,6,9-tetraone, and a material described as bis-tetraaminobenzoquinone^13–22^ and abbreviated as TDT,^13–18^ BTABQ,^18–20^ or TAQ,^21,22^ see Scheme 1. Their syntheses from the shared precursor TABQ, 2,3,5,6-tetraamino-1,4-benzoquinone, are summarized in Scheme 2 and described in more detail in the Materials and Methods section. These materials can compete with inorganic cathode materials in terms of specific and volumetric energy density.^20–22^ They are composed of earth-abundant elements, while common cathode materials such as NCA, NMC, and LCO are disadvantaged by the scarcity of cobalt and the expense of mining and production. Since a reviewer of this work claimed that the materials investigated in this work were of little general interest and “not commonly used”, we feel compelled to point out that “TAQ” was featured in a full-page article in *Chemical & Engineering News* in 2024,^33^ patents have been filed for TAPT^34^ and “TAQ”,^35^ “TAQ” has been licensed to a well-known automobile manufacturer^36^ and it is produced by a *C&EN*-featured^37^ start-up company.

**Scheme 1:**
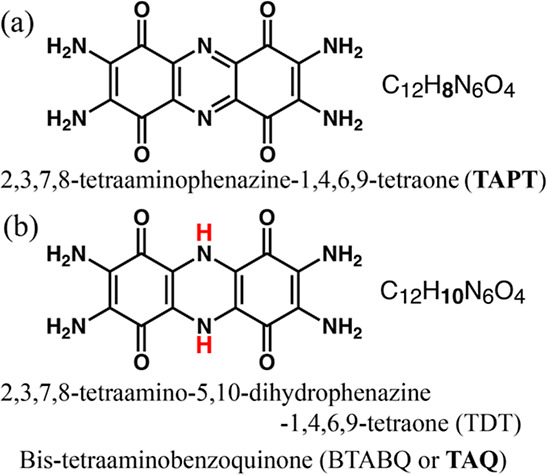
Structures of (a) TAPT^9–13,18^ and (b) TAQ,^21,22^ Also Known as TDT^13–18^ or BTABQ^18–20^ ^a^

a They differ by the hydrogen atoms highlighted in red, which make the central ring pronouncedly antiaromatic,^38^ see Figure S1. The two molecules are neither isomers nor different protonation states of the same structure. The calculated HOMO–LUMO gaps of TAPT and TAQ differ by nearly 1 eV.^21,22^ Note also the different numbers of reducible C=O and N lithium binding sites (6 in TAPT vs 4 in TAQ). In this work, the TAQ molecule as shown in (b) is distinguished from the material previously termed TAQ by quotation marks on the latter; this is necessary because we show here that the material labeled as “TAQ” in the literature does not consist of TAQ molecules.

**Scheme 2:**
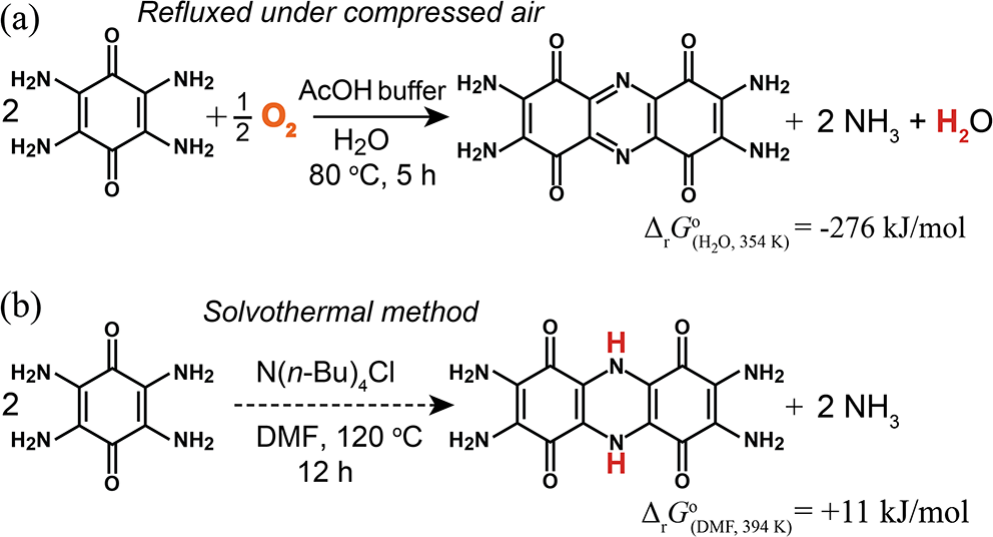
Synthesis of (a) TAPT and (b) TABQ^a^

a The O_2_,^12^ with Δ_r_ *G*° = −225 kJ/mol in the gas phase at 25 °C, Δ_r_ *G*° = −276 kJ/mol in H_2_O at 80 °C, and Δ_r_ *G*° = −249 kJ/mol in DMF at 120 °C, according to quantum chemical calculations and consistent with the high chemical energy of O_2_ with its relatively weak double bond;^39^ (b) Hypothetical synthesis of TAQ from TABQ^21^ with predicted Δ_r_ *G*° = +6 kJ/mol in the gas phase at 25 °C, Δ_r_ *G*° = +14 kJ/mol in H_2_O at 80 °C, and Δ_r_ *G*° = +11 kJ/mol in DMF at 120 °C.

Usually, the chemical structure of small organic molecules like TAPT and “TAQ” would be reliably determined by solution nuclear magnetic resonance (NMR), but due to their insolubility this has not been possible.^21^ Routine ^13^C solid-state NMR (ssNMR) after cross-polarization from ^1^H has been performed on TAPT^9^ and “TAQ” ^16,20,21^ but confoundingly showed four peaks of roughly similar intensities, rather than the expected three peaks from the C=O, C–NH_2_, and =C–N sites (see Scheme 1). NMR of TAPT is challenging because its core is devoid of hydrogen, see Scheme 1a, and therefore conventional cross polarization from ^1^H results in strongly distorted ^13^C and ^15^N NMR peak intensities. The four-peak ^13^C ssNMR peak spectrum has been interpreted in terms of significantly different chemical structures;^9,14,16,20,21^ to resolve these contradictions is our main goal.

Since routine NMR has failed, here we use advanced multinuclear ssNMR and mass spectroscopy, in conjunction with quantum-chemical chemical-shift and energy calculations, to accurately determine the chemical structure of “TAQ” and compare it with TAPT. Chemical bonding is analyzed comprehensively through nearly quantitative fully relaxed direct-polarization or multiCP ^13^C and ^15^N ssNMR; ^13^C­{^1^H} and ^15^N­{^1^H} dipolar dephasing ssNMR experiments to determine ^13^C–^1^H and ^15^N–^1^H proximities; and 2D ^1^H–^13^C ssNMR to detect ^1^H chemical shifts indicative of hydrogen bonding. In addition to unlabeled TAPT and “TAQ”, an expert at MIT synthesized ^15^N_6_-“TAQ” from ^15^N_4_-TABQ. The latter also serves as a model compound for documenting the effects of symmetry-breaking packing effects and hydrogen bonding on ^13^C and ^15^N chemical shifts. The ^15^N enrichment provides quantitative ^15^N spectra of ^15^N_6_-“TAQ” exhibiting an excellent signal-to-noise ratio; with ^15^N­{^1^H} dipolar dephasing it allows for accurate assessment of hydrogen substitution of nitrogen atoms, which is the distinguishing structural difference between TAQ and TAPT, see Scheme 1. It also enables 2D ^15^N–^15^N ssNMR to test for proximity of amine groups. Our multiCP ^15^N NMR spectrum of unlabeled indanthrone, a seven-ring heterocycle whose hydrophenazine core resembles TAQ, indicates that high-quality ^15^N NMR of TAQ would be feasible even without isotope enrichment. Spin–lattice relaxation is fast and nonexponential for ^13^C and ^15^N, and its correlation with linewidths is investigated, since both are affected by persistent radicals.^40,41^ As a result, quite unexpectedly, we show that direct-polarization ^15^N NMR is the best method for obtaining quantitative ^15^N NMR spectra of ^15^N_6_-“TAQ”.

Direct comparison of the spectra of TAPT, whose structure is not controversial and confirmed here, with those of “TAQ”, leads to unambiguous conclusions regarding their structural similarity. The approach of comparing spectra of TAPT and “TAQ” was also taken with MALDI-TOF mass spectrometry. The favorability of TAPT vs TAQ formation is analyzed using quantum chemistry and the instability of TAQ against tautomerization to a reduced from of TAPT is explored. The antiaromaticity of the rings in TAQ and TAPT is probed by nucleus-independent chemical shift (NICS) scans. We comment on the inadvertent duplicative publication of properties of what we show to be the same material, and point out the impact of incorrect “composition of matter” on patent validity.

## Materials and Methods

### Materials

Tetraamino-phenazine-1,4,6,9-tetrone (TAPT) and “TAQ” were synthesized by Bowen Tan in the laboratory of Mircea Dincă at MIT according to published procedures reviewed below,^9,21^ from regular tetraaminobenzoquinone (TABQ) or from ^15^N_4_-TABQ, as outlined in Scheme 2. The synthesis of ^15^N_4_-TABQ is described in the Supporting Information (see Scheme S1). Indanthrone (CAS 81-77-6) was purchased through Fisher Scientific.

### Synthesis of TAPT from TABQ

TAPT was synthesized from TABQ as follows^9^ (see also Scheme 2b): TABQ (0.31 g, 1.8 mmol, 1 equiv) and concentrated hydrochloric acid (0.6 mL, 7.2 mmol, 4 equiv) in H_2_O (17.8 mL) were combined and set to sonicate in a 50 mL round-bottom flask for 15 min. Sodium acetate (1.18 g, 14.4 mmol, 8 equiv) was then added, followed by refluxing at 80 °C under a stream of compressed air for 5 h. The reaction was removed from heat, allowed to cool and filtered through a fine fritted glass filter and rinsed with enough water till filtrate ran clear. Product dried under vacuum for 72 h, a black solid, was isolated (0.20 g, 0.67 mmol, 37% yield).

### Synthesis of “TAQ” and ^15^N_6_-“TAQ” from TABQ

The following describes the synthesis of the “TAQ” and ^15^N_6_-“TAQ” materials from TABQ; an overview of the synthesis is shown in Scheme 2a. A PTFE-capped pressure tube was charged with regular or ^15^N_4_-TABQ (200 mg, 1.2 mmol, 1 equiv), tetrabutyl ammonium chloride (1.30 g, 4.8 mmol, 4 equiv), and DMF (11.4 mL) at ambient temperature and atmosphere. The tube was sonicated for 1 min before heating in an isothermal oven at 120 °C for 12 h. The resulting mixture was filtered and washed with DMF till filtrate was colorless, further washed with methanol, and dried under vacuum, yielding “TAQ” (65 mg, 33% yield).

### Solid-State NMR: General Parameters

Solid-state NMR experiments were performed on a Bruker Avance Neo 400WB spectrometer at resonance frequencies of 400 MHz for ^1^H, 100 MHz for ^13^C and 40.5 MHz for ^15^N, with a Bruker 4 mm magic-angle spinning (MAS)^44^ double resonance probe head. 40 to 60 mg of each sample was packed into a 4 mm outer-diameter zirconia rotor with a 3 mm tall cylindrical glass spacer at the bottom and a Kel-F cap at the top. The 90° pulse durations were 3.6 μs for ^1^H, 4 μs for ^13^C and 7 μs for ^15^N. Proton decoupling was conducted with TPPM at |γ*B*_1_|/2π = 95 kHz before detection and with SPINAL-64 ^45^ at |γ*B*_1_|/2π = 85 kHz during detection. The ^13^C chemical shifts were externally referenced to TMS using the ^13^COO resonance of α-glycine at 176.49 ppm as a secondary reference. The ^15^N chemical shifts were referenced to liquid ammonia via the ^15^N resonance of ^15^N-*t*-Boc-proline at 103 ppm. In all experiments, rotation-synchronized Hahn echoes were generated prior to detection to prevent pulse dead-time baseline distortions. Pulse sequences used in this work for advanced NMR as described in the following sections are compiled in Scheme S2.

### Quantitative ^13^C and ^15^N NMR

Quantitative ^13^C spectra were collected at 14 kHz MAS with direct polarization (DP), using long recycle delays ranging from 50 to 1200 s, with measurement times of 2 days each. DP spectra with shorter recycle delays were also measured to constitute ^13^C saturation recovery series (see Figure S2).

^15^N spectra of all samples studied were acquired at 7 kHz MAS using composite-pulse multiple cross polarization (multiCP) with 2 blocks of 1.1 ms of ramped CP separated by a repolarization time of half the recycle delay, which ranged from 1 to 10 s. Unlike the spectra of TABQ and indanthrone with their NH_*n*_ groups, these spectra of TAPT and “TAQ” were not quantitative because of the lack of protons from the center of the molecule; but DP ^15^N NMR of ^15^N_6_-“TAQ” was quantitative, see below. Measurement times of 1.5 to 8 days were used for the “TAQ” and TAPT samples without ^15^N enrichment. Natural-abundance ^15^N spectra of a tautomer of TABQ and of indanthrone were obtained within one and three days, respectively, while spectra of ^15^N_4_-TABQ in two protonation states were obtained within 10 h. Corresponding spectra of mobile and nonprotonated ^15^N in “TAQ”, indanthrone, and TABQ were obtained after ^1^H–^15^N dipolar dephasing; the selectivity was achieved through turning off the ^1^H decoupling during periods of 4*t*_r_ before and after the π pulse of the rotation-synchronized Hahn echo (i.e., for 1.14 ms in total).

DP spectra with a series of recycle delays and up to 128 scans each were measured on ^15^N_6_-“TAQ” to constitute ^15^N saturation recovery series. Fully relaxed direct-polarization ^15^N NMR spectra of ^15^N_6_-“TAQ” with recycle delays of 50 or 100 s were measured with and without any ^1^H decoupling, both at 7 and 14 kHz MAS, with measurement times of about 12 h each.

### Recoupled Long-Range ^1^H–^13^C Dipolar Dephasing

Recoupled long-range ^1^H–^13^C dipolar dephasing^46^ experiments were conducted at 7 kHz MAS to assess the average dipolar coupling of nonprotonated ^13^C to ^1^H. MultiCP with 10 repolarization delays was used to excite the ^13^C polarization, followed by *N* = 2–8 rotation periods of dipolar recoupling. Spinning sidebands were suppressed by four-pulse total suppression of sidebands (TOSS)^47^ before detection. Corresponding reference experiments (yielding *S*_0_ spectra) with full ^1^H decoupling instead of the recoupling pulses were also conducted to correct for *T*_2C_ relaxation. Measurement of a series took 2 to 3.5 days.

### ^1^H–^13^C Heteronuclear Correlation (HetCor) NMR

^1^H chemical shifts can be probed conveniently in two-dimensional (2D) ^1^H–^13^C and ^1^H–^15^N HetCor NMR experiments, which were conducted at 14 kHz and 7 kHz MAS, respectively, with frequency-switched Lee–Goldburg homonuclear decoupling, recycle delays of 2–10 s, and 0.4 ms of CP. Experiment time was 18 h for “TAQ” and 2 days for TAPT.

### 2D Exchange ^15^N NMR

With two or more spectrally resolved ^15^N-enriched amines in one molecule, 2D exchange ^15^N NMR can address the question if ^15^NH_2_ groups of different chemical shifts are *ortho* to each other. If this is case, dipolar ^15^N spin exchange during a mixing time of a few seconds will produce an off-diagonal cross peak. The experiment was demonstrated on ^15^N_4_-TABQ as a model compound and successfully applied to ^15^N_6_-“TAQ” at 7 kHz and 4 kHz MAS. ^15^N signal was generated by 0.4 ms of cross polarization from ^1^H. The maximum evolution time was 9 ms. With a mixing time of 0.02 s, a diagonal spectrum is quickly obtained for reference, while significant cross peaks are observed after 2 s. Longer mixing times would increase the measurement time and decrease the signal by *T*_1N_ relaxation. The combined experiment time for the two experiments on ^15^N_6_-“TAQ” was 18 h.

### CODEX ^15^N NMR

In order to rule out a model of intermolecular ^15^N spin exchange proposed by a reviewer, we performed CODEX ^15^N NMR on ^15^N_6_-“TAQ” at 4 kHz MAS, for a series of mixing times from 0.1 to 2 s. Recoupling times *Nt*_r_ of 1.5 and 2 ms were used to show that the dephasing was in the long-time limit. The series required 16 h of signal averaging.

### MALDI-TOF Mass Spectrometry

Matrix-assisted laser-desorption ionization time-of-flight mass spectrometry was conducted on TAPT and “TAQ” using a Voyager DE-Pro instrument in linear mode. The Super-DHB (Millipore Sigma, 50862-1G-F) matrix was prepared fresh for analysis as a 20 mg/mL solution in a 50:50 (v/v) mixture of acetonitrile and RNase-free water with 0.1% TFA. Powder samples were suspended to a concentration of 1.67 mg/mL of acetonitrile, left to settle, pipetted from the bottom of the scintillation vial, mixed 1:1 with the matrix and spotted onto a 100-spot stainless steel plate. Dihydroxybenzoic acid (*m*/*z* = 137.02332 Da for M + H–H_2_O^+^ and 177.01583 Da for M + Na^+^) in the matrix was used for calibration.

### Quantum Chemistry

Quantum-chemical calculations were performed using Gaussian 16.^43^ Clusters of TAPT and TABQ were extracted from the published crystal structures,^20,48^ hydrogen atoms were added and then were geometrically optimized with M062X/6-31+G­(d,p). The magnetic shieldings of the optimized clusters were calculated with mPW1PW91/6-311+G­(2d,p) using the gauge including atomic orbitals (GIAO) method. The isotropic ^15^N magnetic shielding σ_iso_ was converted to chemical shift δ_iso_ using the equation δ_iso_ = (238.61 ppm– σ_iso_)/1.0429. The ^13^C magnetic shielding values were converted to chemical shifts using scaling factors published by Lodewyk et al.,^49^ specifically δ_iso_ = (185.6582 ppm– σ_iso_)/1.0221. Gibbs free energies of reactions were calculated with CBS-4M. Some of the calculations were performed in implicit solvent using the SMD continuum density solvation model.^50^

In order to probe (anti)­aromaticity, nucleus independent chemical shifts (NICS) were calculated with mPW1PW91/6-311+G­(2d,p) using GIAO. The phantom atoms were placed 1.7 Å above the molecules, in step sizes of 0.2 Å along the long axis of the fused ring molecules. The negative of the *zz* component of the magnetic shielding tensor of the phantom atom was taken as the NICS value.^51^

## Results

### ^13^C NMR Spectra

Nearly quantitative ^13^C NMR spectra of TAPT and two “TAQ” samples obtained by direct polarization after nearly full relaxation are compared in Figure 1. All samples show two relatively narrow peaks near 175 ppm and 147 ppm, and a doublet centered on 133 ppm, in approximate 1:1:(0.5 + 0.5) ratios (see also Figure S3). The positions and relative intensities of the peaks are in good agreement among the three samples. Predicted chemical shifts of TAPT, marked by vertical lines in Figure 1a, match experimental peak positions within ±4 ppm. This adequate agreement validates the predictions since the structure of TAPT is not contentious. By contrast, most of the predictions for “TAQ” assuming the structure of TAQ from Scheme 1b are far off, both for the isolated molecule and with crystal-packing effects, in some cases by ≥19 ppm, see Figure 1b,c.

**Figure 1:**
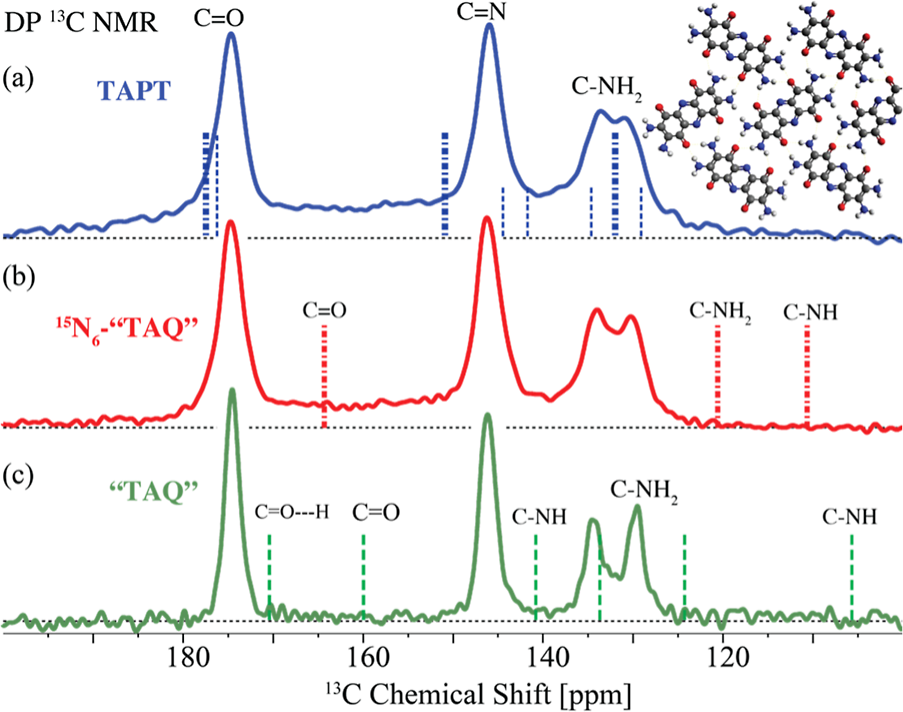
Quantitative direct-polarization ^13^C NMR spectra of (a) TAPT, (b) ^15^N_6_-“TAQ”, and (c) “TAQ” measured at 14 kHz MAS with recycle delays of 100 s, 100 s, and 1200 s, respectively. The same chemical-shift pattern is observed. Chemical-shift predictions for TAPT in (a) and TAQ in (b,c) obtained by empirical ACD/NMR^42^ for single molecules are marked by thicker dash-dotted lines, three per molecule. Quantum-chemical predictions by Gaussian^43^ for crystalline hydrogen-bonded molecules (see the inset) are indicated by thin dashed vertical lines, five or six per molecule due to symmetry-breaking crystal-packing effects.

^13^C NMR spectra of TAPT and “TAQ” can also be obtained by ^1^H–^13^C cross polarization.^9,14,16,20–22^ Figure S3 compares DP and multiCP ^13^C NMR spectra of “TAQ” and lists peak integrals. The spectra are similar, with multiCP providing a better signal-to-noise ratio, as usual.^52^ The carbon resonating near 147 ppm is difficult to cross polarize, which indicates that it is far from the nearest proton; this is confirmed by ^13^C­{^1^H} NMR below. In previously published cross-polarization ^13^C NMR spectra of “TAQ” and TAPT,^9,14,16,20,21^ this central peak was at least 2-fold underrepresented, which interfered with the interpretation of the spectra.

In Figure 1, linewidths differ between samples; this can be attributed to differences in the concentration of unpaired electrons, as confirmed below. Note that the line width and the distance between the two right-most peaks of ^15^N_6_-“TAQ” is intermediate between that in “TAQ” and TAPT, indicating a continuum of physical properties for the same underlying chemical structure, rather than a structural distinction between TAPT and “TAQ”.

### ^13^C and ^1^H Spin–Lattice Relaxation

The nearly quantitative direct-polarization ^13^C NMR spectra of TAPT and “TAQ” shown in Figure 1 were obtained with moderate recycle delays based on the relatively fast *T*_1C_ relaxation in these materials, which is unexpected for such rigid, hydrogen-bonded solids. To document the fast relaxation and shed light on its origin, Figure 2a presents ^13^C saturation-recovery intensity data for the three samples; the underlying series of spectra of TAPT and ^15^N_6_-“TAQ” are shown in Figure S2. The observed relaxation is nearly uniform across all peaks and nonexponential. For TAPT and ^15^N_6_-“TAQ”, the peak intensity at time *t* can be fit with $1-\exp\left(-\sqrt{t/\tau_{1}}\right)$ , see Figures 2a and S4a, which is characteristic of relaxation by randomly distributed unpaired electrons (see the derivation in the Supporting Information). The time constant τ_1_ is approximately 5 s. For “TAQ”, the fit curve contains a product of $1-\exp\left(-\sqrt{t/\tau_{1}}\right)$ and regular exponential relaxation. More typical slow and exponential relaxation of the 178 ppm peak of TABQ is also shown.

**Figure 2:**
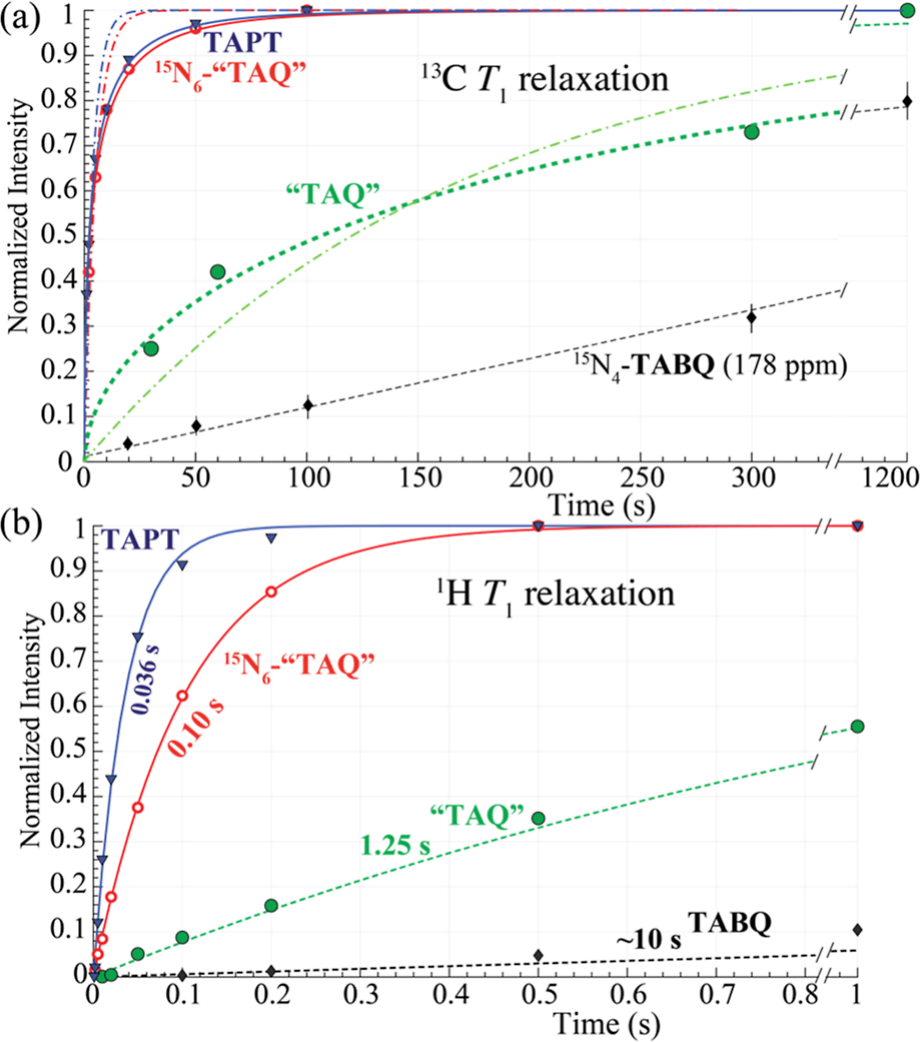
Spin–lattice relaxation of TAPT (blue triangles), ^15^N_6_-“TAQ” (open red circles), “TAQ” (filled green circles), and TABQ (filled black diamonds). (a) Signal intensity in ^13^C saturation-recovery as a function of recovery time; underlying series of spectra are shown in Figure S2. Solid fit curves are of the type (1 – exp­(−(*t*/τ)^1/2^)), typical of relaxation due to randomly distributed unpaired electrons^53,54^ (as derived in the Supporting Information), while dash-dotted curves are poor exponential fits requested by a reviewer (see also Figure S4a). The dashed fit curve for “TAQ” combines this with regular exponential relaxation. (b) ^1^H saturation recovery data, obtained with probe-head background suppression; all are fitted by conventional (inverted decreasing) exponential functions.

Figure 2b shows ^13^C-detected ^1^H saturation recovery data of TAPT, two “TAQ” samples, and TABQ, while Figure S4 documents ^15^N saturation recovery of ^15^N_6_-“TAQ”. Spin–lattice relaxation is very fast for TAPT and ^15^N_6_-“TAQ”, e.g. with *T*_1H_ on the 50 ms time scale, and moderately fast for “TAQ”, while TABQ shows the slow relaxation expected for a rigid molecular crystal. This unusual relaxation behavior matches the line width trend in Figure 1 and can only be explained in terms of the local magnetic fields of randomly distributed unpaired electron spins (i.e., persistent radicals). Indeed, a strong EPR signal has been reported for “TAQ”.^16^ Note that variations in the fairly low density of persistent radicals can account for the difference in relaxation and line width observed between “TAQ” and ^15^N-“TAQ”; similar variations between batches of nominally the same material have been observed before.^40^

### ^15^N NMR Spectra

Figure 3 compares multiCP ^15^N NMR spectra of TAPT and two “TAQ” samples recorded under the same conditions (7 kHz MAS). While the spectrum of TAPT is noisy even after 8 days of signal averaging, its similarity to the other two spectra can still be recognized. Given the universal agreement on the structure of TAPT,^9^ this spectrum is not needed for structure determination, but only as a reference in the analysis of the contentious structure of “TAQ”. The spectrum of ^15^N_6_-“TAQ” in Figure 3b, with an excellent signal-to-noise ratio due to the ^15^N enrichment, exhibits a doublet near 70 ppm, a sharp peak at 309 ppm, and a spinning sideband of the latter near the center of the spectrum. The assignment as a sideband is confirmed by its absence from the 14 kHz MAS spectrum in Figure 4. The same peak pattern is observed for unlabeled “TAQ” in Figure 3c. As in ^13^C NMR, the line width is largest for TAPT and smallest for “TAQ”, while that of ^15^N_6_-“TAQ” is intermediate. The observed chemical shifts are in adequate agreement with values predicted for TAPT but not TAQ.

**Figure 3:**
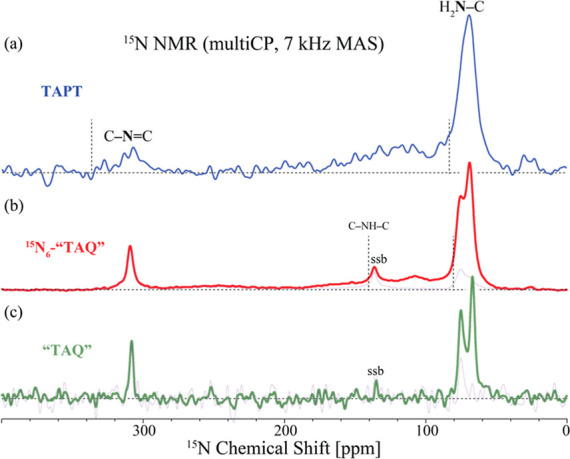
MultiCP ^15^N NMR spectra of (a) TAPT and (b,c) two “TAQ” samples recorded at 7 kHz MAS, with ACD/NMR database predicted peak positions for TAPT and TAQ marked as vertical dashed lines. Thin purple lines in (b,c): spectra after dipolar dephasing. “ssb”: spinning sideband.

**Figure 4:**
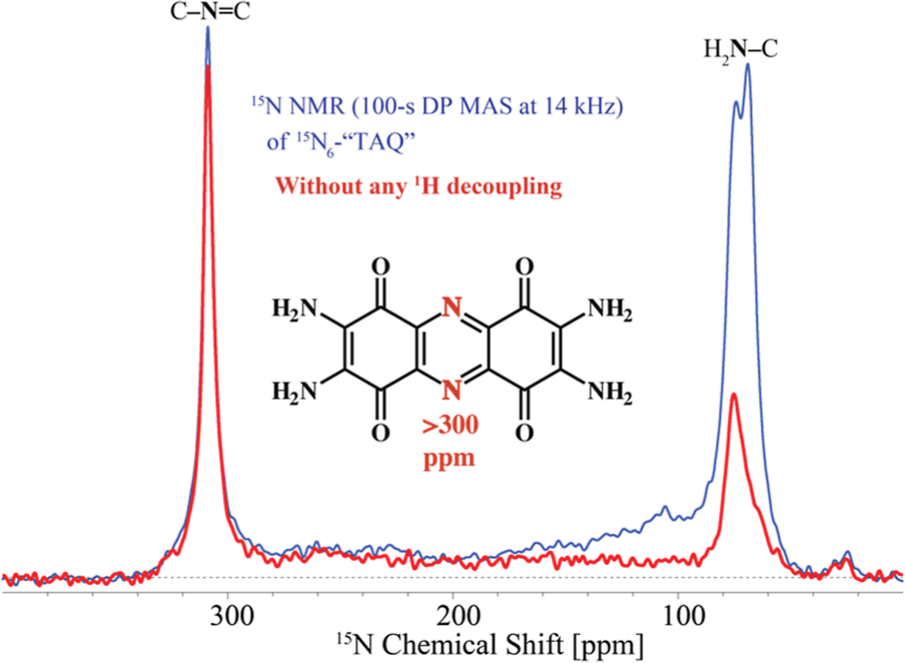
Probing the bonding of the central nitrogen atoms in ^15^N_6_-“TAQ” by ^15^N NMR and ^15^N­{^1^H} dipolar dephasing. Thin blue line: quantitative, fully relaxed direct polarization ^15^N NMR spectrum of ^15^N_6_-“TAQ”, recorded at 14 kHz MAS with a 100 s recycle delay. Thick red line: corresponding spectrum (unscaled) without any ^1^H decoupling after 1.1 ms of dipolar dephasing and ^15^N *T*_2_ relaxation, proving that N resonating at 309 ppm is not bonded to H. Two fairly small spinning sidebands of the peak at 309 ppm, at 653 ppm and −36 ppm, are outside the spectral range shown.

### Quantitative ^15^N NMR of ^15^N_6_-“TAQ”

Figure 4 shows a nearly quantitative ^15^N NMR spectrum of ^15^N_6_-“TAQ” obtained by direct polarization with 100 s recycle delay at 14 kHz MAS. Like ^13^C nuclear spins in TAPT and “TAQ”, ^15^N nuclear spins in ^15^N_6_-“TAQ” undergo surprisingly fast spin–lattice relaxation, see Figure S4, making quantitative DP ^15^N NMR possible. The ^15^N spectrum shows the 309 ppm peak very prominently; spinning sidebands outside of the spectral range shown contain an additional 16% of the intensity. This demonstrates that the 309 ppm peak accounts for about 1/3 of all nitrogen sites, while in the multiCP spectra of Figure 3 it is reduced in intensity due to slow cross polarization from ^1^H and because about half of the total intensity is dispersed into the spinning sidebands at the lower spinning frequency of 7 kHz.

### ^15^N NMR without ^1^H Decoupling

Also prominently included in Figure 4 (in red) is a spectrum of ^15^N_6_-“TAQ” recorded under nearly the same conditions except for an added 1.1 ms ^15^N *T*_2_ filter and no ^1^H decoupling. Little intensity loss or broadening of the line at 309 ppm results from the absence of ^1^H decoupling (unlike, for instance, in ^15^N-*t*-Boc-proline), which indicates that this is a signal of nitrogen not bonded to hydrogen—actually, it must be far from the nearest protons. This most directly rules out the TAQ structure with its hydrogen-substituted central nitrogen atoms, see Scheme 1b.

To document that the lack of dephasing or line broadening is not due to fast MAS, the corresponding pair of spectra has also been recorded at 7 kHz MAS, as shown in Figure S5. Again, the lack of ^1^H decoupling does not significantly affect the nitrogen resonating at 309 ppm, confirming a long distance from the nearest protons.

### Long-Range ^1^H–^13^C Dephasing

The proximity of specific ^13^C sites to protons in “TAQ” can be probed by recoupled ^13^C­{^1^H} dipolar dephasing^46^ and compared with dephasing in TAPT. Figure 5 presents spectra of TAPT and “TAQ” after 0.858 ms of recoupled dephasing, while series of spectra are shown in Figure S6. The resonance of the nitrogen-bonded carbon at 147 ppm exhibits the slowest dephasing in both samples. Indeed, in the known structure of TAPT (see inset in Figure 5b), the C=N carbon is separated by four bonds, or about 4 Å, from the nearest protons. This carbon dephases more slowly than any carbon in the anthraquinone model compound^46^ in Figure S7.

**Figure 5:**
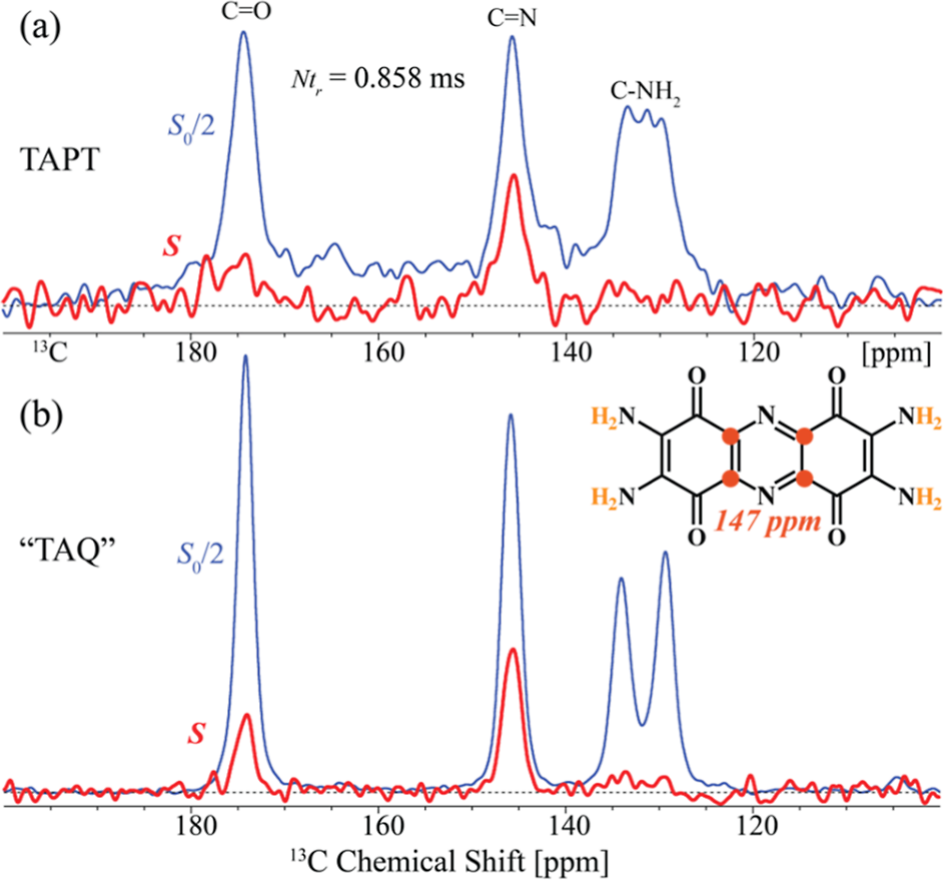
Probing proximity of ^13^C to ^1^H by long-range ^13^C­{^1^H} dephasing. Shown are ^13^C NMR spectra (in red) of (a) TAPT and (b) “TAQ” after dephasing for 0.858 ms, measured at 7 kHz MAS. In both comparisons, the reference spectrum *S*_0_ (in blue) has been scaled down by a factor of 0.5 for easier comparability.

### Amine NMR Doublets in Solid TABQ

The solid-state ^13^C and ^15^N NMR spectra of “TAQ” and TAPT each show one more peak than expected, due to a doubling of the ^15^NH_2_ signals near 70 ppm and the ^13^C–NH_2_ signals near 133 ppm. We document here that this is a normal result of symmetry breaking in the solid state, due to differences in hydrogen bonding (see the inset in Figures 1a and S8), using the simple, highly symmetric, and soluble TABQ molecule (see Figure 6 and the crystal structure in Figure S9) as a model compound with only two chemically distinct types of carbons, C=O and C–NH_2_. Figure 6 compares the ^13^C and ^15^N solid-state NMR spectra of ^15^N_4_-TABQ, which show substantial splittings of the ^15^NH_2_ and ^13^C–NH_2_ signals, with the solution NMR spectra, where the splittings disappear as expected.

**Figure 6:**
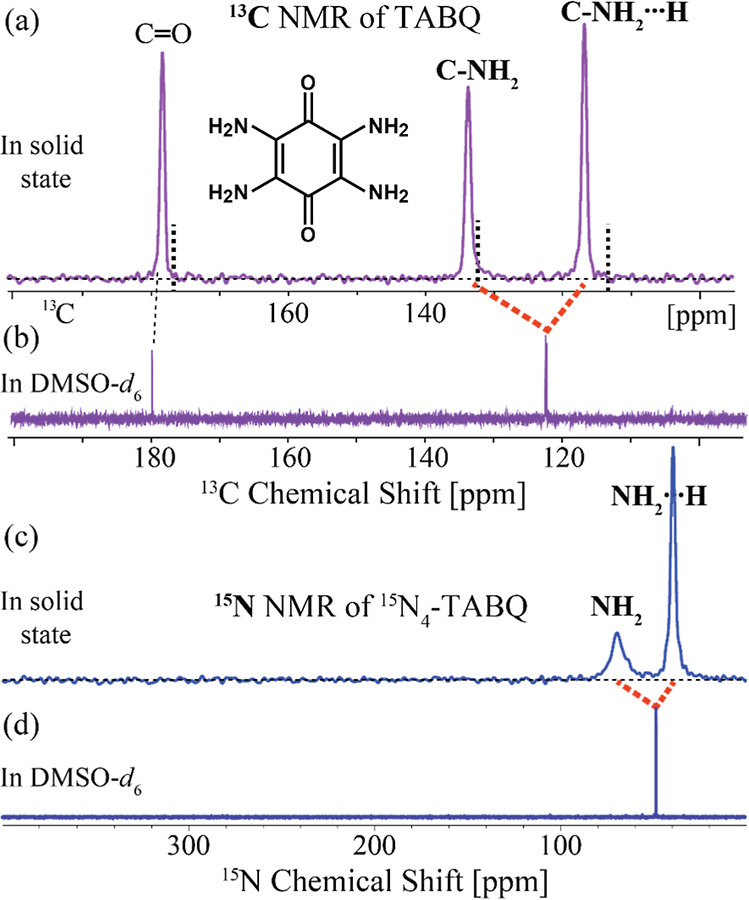
Packing-induced symmetry breaking in TABQ in the solid state as seen in (a,b) ^13^C and (c,d) ^15^N NMR. (a,c) Solid-state multiCP ^13^C and ^15^N NMR spectra of ^15^N_4_-TABQ. (b) Solution ^13^C NMR spectrum of TABQ. (d) ^15^N NMR spectrum of 17 mg ^15^N_4_-TABQ in 0.4 mL DMSO with a 50 s recycle delay. ^13^C chemical shifts predicted by optimized quantum-chemical simulations with hydrogen bonding (see structure in Figure S9a) are marked as dashed vertical lines. In the “C–NH_2_···H” moiety, the nitrogen atom of the amine group is the hydrogen-bond acceptor.

Note that in “TAQ” and TAPT the splittings are actually smaller than those in the TABQ model system, showing that the splittings observed for the electrode materials are not unusual. Quantum-chemical calculations indicate that the C–NH_2_ splittings in the ^15^N and ^13^C solid-state NMR spectra of TABQ are due to differences in hydrogen bonding of the primary amines, with the right-most peak in either spectrum assigned to a hydrogen-bond-accepting nitrogen atom of an amine. The crystal structure of TAPT in Figure S8b shows only N–H···O hydrogen bonds; one NH_2_ group is a double, the other a single hydrogen-bond donor. This smaller structural difference is consistent with the smaller chemical-shift difference, compared to TABQ.

### 2D ^15^N NMR of ^15^N_6_-“TAQ”: Proximity of the ^15^NH_2_ Sites

The preceding analysis suggests that ^15^NH_2_ with different chemical shifts may be found in the same molecule. This assumption can be confirmed by 2D exchange ^15^N NMR. If nitrogen nuclei with different chemical shifts are proximal to each other, transfer of ^15^N magnetization by the interamine ^15^N–^15^N dipolar coupling results in a frequency change that produces off-diagonal cross peaks in the 2D spectrum. We demonstrated this principle on ^15^N_4_-TABQ with its well-resolved amine peaks, see Figure 7a, and then applied the 2D experiment to ^15^N_6_-“TAQ”, which also gives cross peaks after *t*_m_ = 2 s, which show that the resolved NH_2_ peaks in “TAQ” are from neighboring (*ortho*) amines.

**Figure 7:**
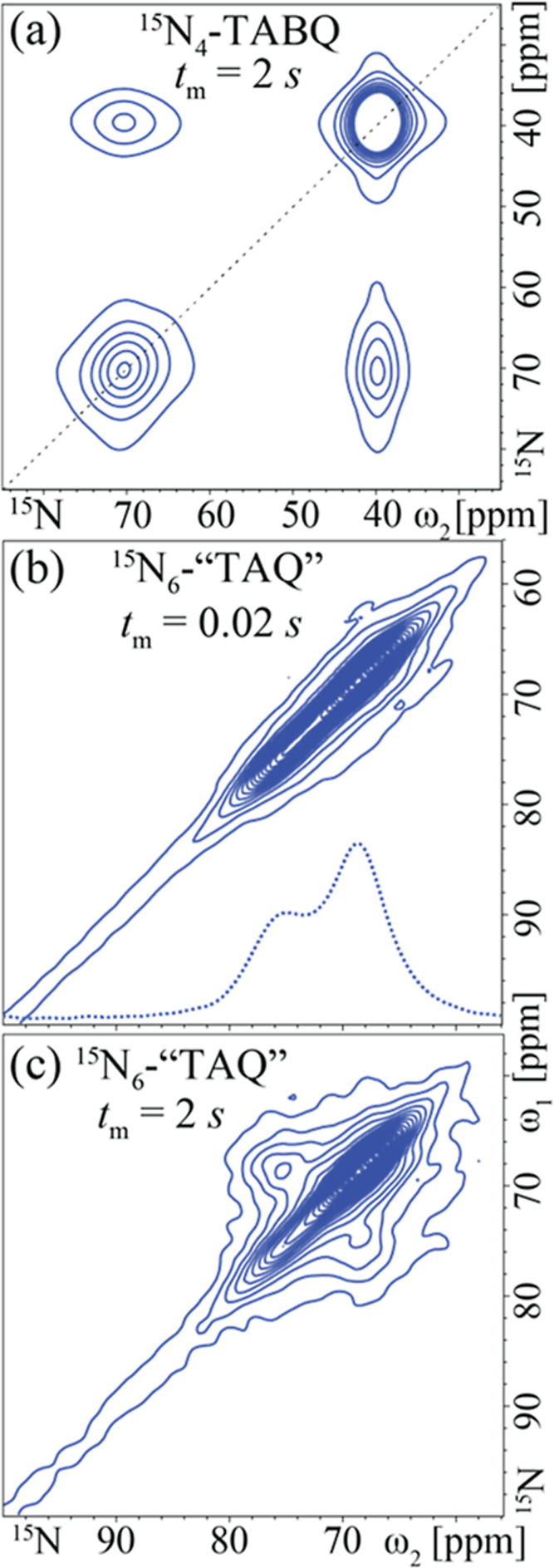
Probing the proximity of differently hydrogen-bonded amines by two-dimensional exchange ^15^N NMR of (a) ^15^N_4_-TABQ and (b,c) ^15^N_6_-“TAQ” after (b) a short 0.02 s and (a,c) a 2 s mixing time. The off-diagonal peaks in (a,c) document the proximity of the spectrally resolved amines.

Cross peaks predominantly from intermolecular magnetization transfer across a hydrogen bond are unlikely given that the intramolecular *ortho* N–N distance is always favorably small at 2.8 Å, while the shortest intermolecular N–N distances in TAPT are 3.7 Å and 4.6 Å. As a result, the intramolecular N–N dipolar coupling is at least twice larger than any intermolecular one. A model with intermolecular cross peaks, while the *ortho* NH_2_ groups are chemically equivalent, see Figure S8, and therefore do not produce intramolecular cross peaks, was ruled out by ^15^N centerband-only detection of exchange (CODEX) NMR,^55^ see Figure S8a. In brief, CODEX would detect additional much faster intramolecular exchange, but its observed exchange rate is similar as in the 2D experiment, see Figure S8a.

### ^15^N Spectra of Various NH_*n*_ Groups

In order to provide a solid basis for ^15^N chemical-shift analysis of “TAQ”, Figure 8 shows examples of ^15^N NMR signals of several types of NH_*n*_ groups potentially relevant in “TAQ”. Figure 8a shows the multiCP spectrum of nitrogen in the dihydrophenazine core of indanthrone without ^15^N enrichment. Dipolar dephasing (red trace) confirms that the nitrogen atoms are bonded to hydrogen. Why dihydrophenazine is stable in indanthrone but not in TAQ is discussed below. The high-quality ^15^N spectra of the large indanthrone molecule in Figure 8a indicate that even without ^15^N enrichment we would be able to obtain good ^15^N NMR spectra of TAQ, if it existed.

**Figure 8:**
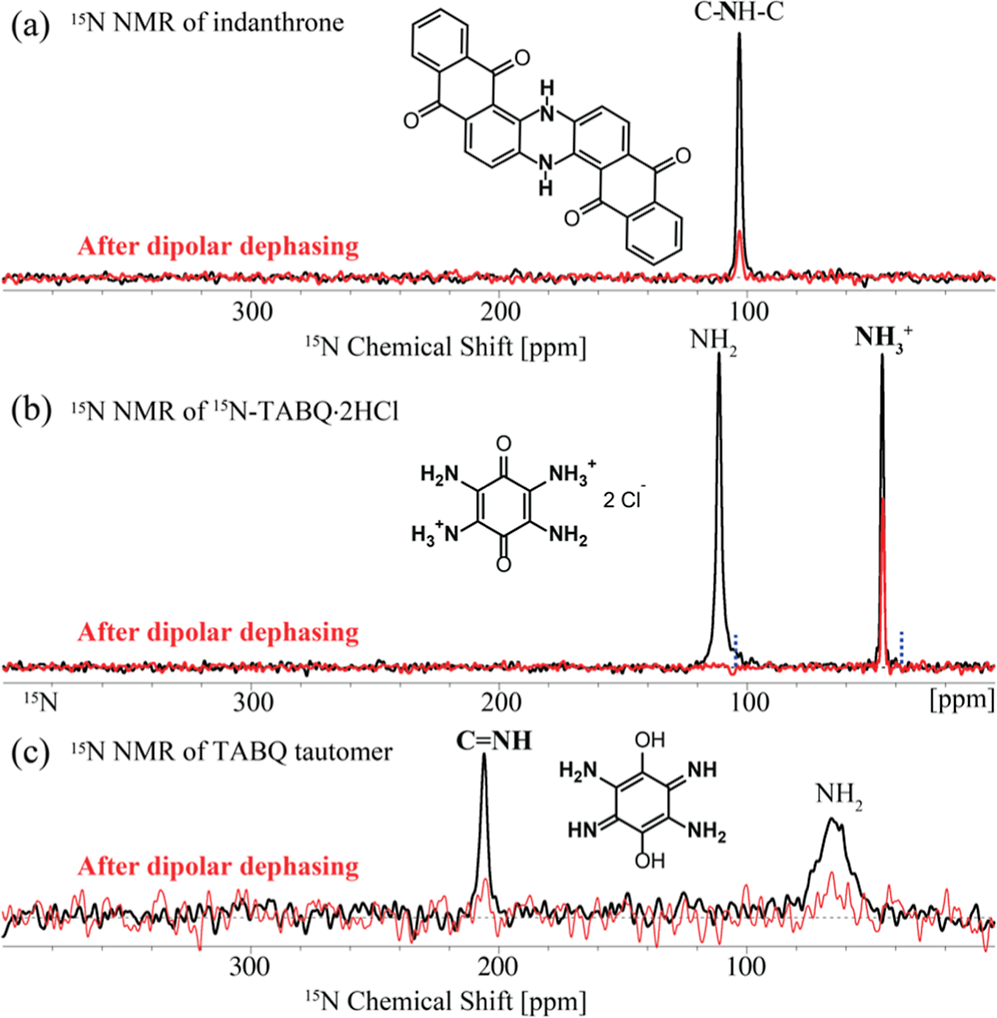
^15^N NMR after multiCP (black traces) and with 1.1 ms of dipolar dephasing added (red traces) of (a) indanthrone with a dihydrophenazine core as in TAQ, (b) TABQ·2HCl with NH_3_^+^ groups, and (c) a tautomer of TABQ with a C=N–H imine. ^15^N chemical shifts predicted by optimized quantum-chemical simulations are marked as dashed vertical lines.

Figure 8b shows how protonation of amine groups of TABQ, in TABQ·2HCl produced by treatment with 6 M HCl, shifts their resonance to the right and the peak of their neighboring nonprotonated NH_2_ groups to the left. The corresponding ^13^C NMR spectrum of TABQ·2HCl, with a shift of the C–NH_3_^+^ resonance to 100 ppm, is shown in Figure S9. Figure 8c documents the 208 ppm resonance of a C=N–H imine in a tautomer of TABQ.

### ^1^H–^13^C Heteronuclear Correlation NMR

^1^H chemical shifts can be obtained with moderately high resolution in 2D ^1^H–^13^C heteronuclear correlation (HetCor) NMR with ^1^H homonuclear decoupling. The 2D spectra of TAPT and “TAQ” are shown in Figure S10. They exhibit ^1^H chemical shifts of about 6.6 ppm. There is no indication of any central protons attached to the pyrazine ring. This contrasts with ^1^H–^13^C HetCor spectra of indanthrone, see Figure S11, which prominently show the resonance of the protons in the dihydrophenazine core.

### Mass Spectrometry

After NMR, mass spectrometry is the next best method for comparing the structures of TAPT and “TAQ”, based on the 2 Da larger mass of TAQ vs TAPT (see Scheme 1). Figure 9 compares the matrix-assisted laser desorption ionization time-of-flight (MALDI-TOF) mass spectra of TAPT and “TAQ”; the similarity of the spectra is pronounced, confirming the structural similarity deduced from the NMR spectra. No systematic shift by +2 Da is observed between the mass spectra of TAPT and “TAQ”.

**Figure 9:**
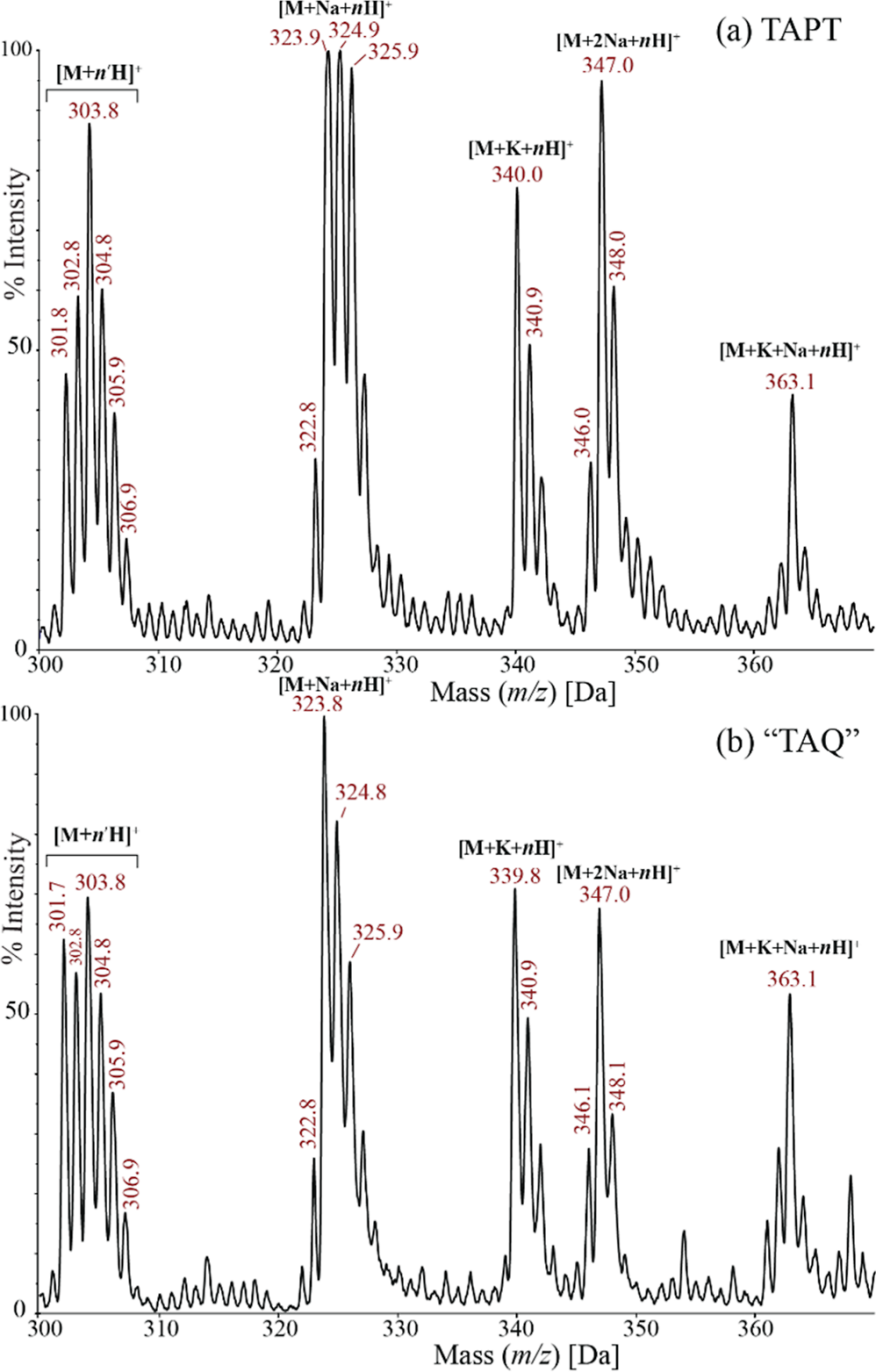
MALDI-TOF mass spectra of (a) TAPT and (b) “TAQ”. Major peaks are labeled with their measured masses in Da.

The sharpest and most prominent peak clusters in Figure 9a can be assigned to sodium and potassium adducts of TAPT, such as [M + Na + *n*H]^+^, [M + K + *n*H]^+^ and [M + 2Na + *n*H]^+^, with *M* = 300.06 Da^56^ for TAPT (C_12_H_8_N_6_O_4_) and small integers *n*. Such alkali adducts are not too surprising with molecules designed to strongly bind alkali atoms in an electrode. The highest peaks are observed for *n* = 1. These distinctive features are directly matched by peaks in the spectrum of “TAQ” in Figure 9b, without a 2 Da shift, indicating that there is no structural difference between TAPT and “TAQ”.

In both mass spectra, wide clusters of [M + *n*′H]^+^ peaks observed slightly above 300 Da can be explained by reduction of TAPT by up to six hydrogen atoms (plus protonation of one NH_2_). These species include radical cations such as [M + 2H]^+•^ previously documented prominently in anthraquinones.^57,58^ The highest peak here corresponds to *n*′ = 4. The mass range spanned by these peaks exceeds the TAQ – TAPT mass difference of 2 Da by more than a factor of 2, which makes this peak cluster less suitable for structure matching.

## Discussion

### Agreement of “TAQ” and TAPT Spectra

The ^13^C NMR, ^15^N NMR, and MALDI-TOF peak positions and intensities of TAPT and “TAQ” are in good agreement. This in itself is sufficient as proof that the two materials have the same chemical structure. Through chemical-shift prediction, mass spectrometry peak assignment, and NMR spectral editing, we have shown that the molecular structure of “TAQ” is that of TAPT. The chemical shifts predicted for TAPT, whose structure is not in dispute, are in adequate agreement with experimental peak positions, which validates the predictions. The ^13^C NMR peak positions of TAPT^9^ and “TAQ” in the literature^21^ agree with ours, confirming the identity of the materials. (The peak intensities in the literature were strong distorted, similarly so for TAPT^9^ and “TAQ”,^21^ because routine CP from ^1^H does not adequately cross polarize the carbon nuclei near the proton-free core of TAPT, see Scheme 1a.)

### The Central Ring in “TAQ”

The structural difference between TAPT and TAQ, see Scheme 1, resides in the central ring. NMR shows multiple lines of strong evidence that “TAQ” contains the same hydrogen-free pyrazine ring as TAPT. It features a pyridinic *N* with a chemical shift of 309 ppm that is not bonded to ^1^H; in fact, this nitrogen atom is so far from the nearest ^1^H that its ^15^N NMR signal remains sharp without any ^1^H decoupling, see Figure 4. Our ^15^N NMR spectra of ^15^N_6_-“TAQ” confirm completely nonprotonated pyrazine ^15^N at 309 ppm not only qualitatively but quantitatively. It is unlike the dihydrophenazine N-H in indanthrone, see Figure 8a, which resonates at a 200 ppm lower chemical shift and undergoes dipolar dephasing.

### Differences in Predicted Properties of TAPT vs TAQ

The two hydrogen atoms attached to the central pyrazine ring of TAQ have pronounced effects on predicted properties. They break up the conjugated π-electron system in TAPT, which increases the calculated HOMO–LUMO gap of TAQ by nearly 1 eV relative to the gap in TAPT.^21,22^ The predicted ^13^C and ^15^N chemical shifts are also very different, see the dashed vertical lines in Figures 1 and 3. Furthermore, the NICS scan in Figure S1 shows that the central ring in TAQ would be strongly antiaromatic. Finally, the central hydrogen atoms in TAQ block two of the six lithium-binding sites in TAPT, reducing the theoretical charge-storage capacity.

### Tautomerism?

Tautomerism has been invoked to explain the ^13^C NMR resonances in “TAQ”,^21,22^ but this is a questionable, incomplete analysis, since the two tautomers would generate more signals than the four observed, see Figure S12. Furthermore, some of the ^15^N and ^13^C chemical shifts predicted for these tautomeric structures are off by more than 50 ppm and 20 ppm, respectively, from the peaks in the experimental spectra. One characteristic of keto–enol tautomerism would be the formation of C=N–H groups, which is distinctive in ^15^N NMR, resonating near 212 ppm, according to ACD/Laboratories, quantum-chemical chemical shift predictions, and the spectrum of a tautomer of TABQ shown in Figure 8c. No such C=N–H ^15^N NMR signal was observed in “TAQ”, see Figure 4.

Tautomerism should not be confused with quantum-mechanical resonance. Unlike resonance structures, different tautomers are distinct, isolatable structures. Tautomerism as a fast dynamic process has been observed in solids only when it involved a short-range (<3 Å) proton transfer;^59^ in solution, complicated structural changes, as implied by Figure S12 (where proton donor and acceptor are separated by more than 3 Å), rely on proton shuttling by the solvent (so it is actually a different proton that leaves one site than bonds to the other). If tautomerism was fast on the NMR time scale, the number of ^13^C NMR peaks would be reduced back to three but their chemical shifts would be lower than observed experimentally because the chemical shifts of the =N–H and O–H containing tautomer in Figure S12 are all too low. In short, tautomerism is rare in the solid state and there are no indications of tautomerism in “TAQ”.

### Symmetry Breaking in the Solid State

TAPT and TABQ each contain four amine groups that are symmetry-equivalent in solution but can have different resonance frequencies in the solid state, for instance if their hydrogen bonding is different. This has been documented here in both ^13^C and ^15^N NMR of TABQ, see Figure 6, and explains the observation of two NH_2_ peaks in ^15^N NMR, and two C–NH_2_ bands in ^13^C NMR, of TAPT and “TAQ”. Quantum-chemical calculations show that the left peak is from the hydrogen-bond donor, contrasting with the very low chemical shifts observed in Figures 8b and S9b for the NH_3_^+^ groups of TABQ·2HCl, which have fully accepted a proton, and for the carbons they substitute. The ^15^NH_2_ and ^13^C–NH_2_ peak splittings are smaller in TAPT than in TABQ, demonstrating that these are ordinary examples of hydrogen-bonding effects on chemical shift.

The combination of packing-induced peak splittings and strongly distorted peak intensities in literature CP spectra of TAPT^9^ and “TAQ”^21^ led to four peaks of ∼2:1:(2 + 2) intensity ratios, instead of the expected three peaks in a 1:1:1 ratio in the solution-NMR spectrum of TAPT. This confounded the analysis of the CP spectra. In this study, by performing nearly quantitative DP and multiCP experiments, see Figures 1 and S3, we have shown that the peak intensities are 2:2:(1 + 1), fully consistent with the structure of TAPT. This highlights the importance of quantitative peak intensities, which are achievable in modern solid-state ^13^C NMR even for a difficult system like TAPT with its hydrogen-deficient core.

### Fast Spin–Lattice Relaxation: Persistent Radicals

The ^13^C and ^15^N NMR spectra of the TAPT and two “TAQ” samples analyzed here show systematically different line widths and broad background between the sharp peaks. Fast spin–lattice relaxation, see Figure 2, provides strong evidence that these are due to different concentrations of unpaired electrons,^40^ i.e., persistent radicals. Most characteristic is the uniform, unusually fast, specifically nonexponential ^13^C relaxation, see Figure 2a, observed for the samples with the largest line broadening. The ^1^H relaxation in Figure 2b is also very fast. Occasional semiquinone forms of TAPT^21^ provide a likely mechanism of radical formation.

### Stability of TAQ vs TAPT

The synthesis of TAPT from TABQ and 1/2 O_2_ ^12^ is highly exergonic, see Scheme 2a, because O_2_ is a weakly bonded, high-energy molecule^39^ and strongly bonded H_2_O forms as a product. By contrast, the Δ_r_ *G*° of TAQ synthesis from TABQ in Scheme 2b is slightly positive (unfavorable), according to quantum-chemical calculations, which is consistent with the fact that the reaction lacks any driving force in terms of bond energies: the numbers of N–H, C–N, and C=O bonds remain unchanged. Formation of the antiaromatic central ring in TAQ^38^ is expected to be unfavorable. This suggests that TAPT will form from TABQ instead of TAQ, given that O_2_ is ubiquitous.

Quantum chemical calculations, as summarized in Scheme 3a, further show quite clearly that TAQ is unstable against a tautomeric shift of two hydrogen atoms from N–H into O–H groups, which eliminates the antiaromaticity^38^ of the central ring of TAQ, see Figure S13, and results in a reduced form of TAPT with a favorable aromatic ring, see Scheme 3a and Figure S13. The predicted Gibbs free energy decrease is substantial, Δ_r_ *G*° = −85 kJ/mol.

**Scheme 3:**
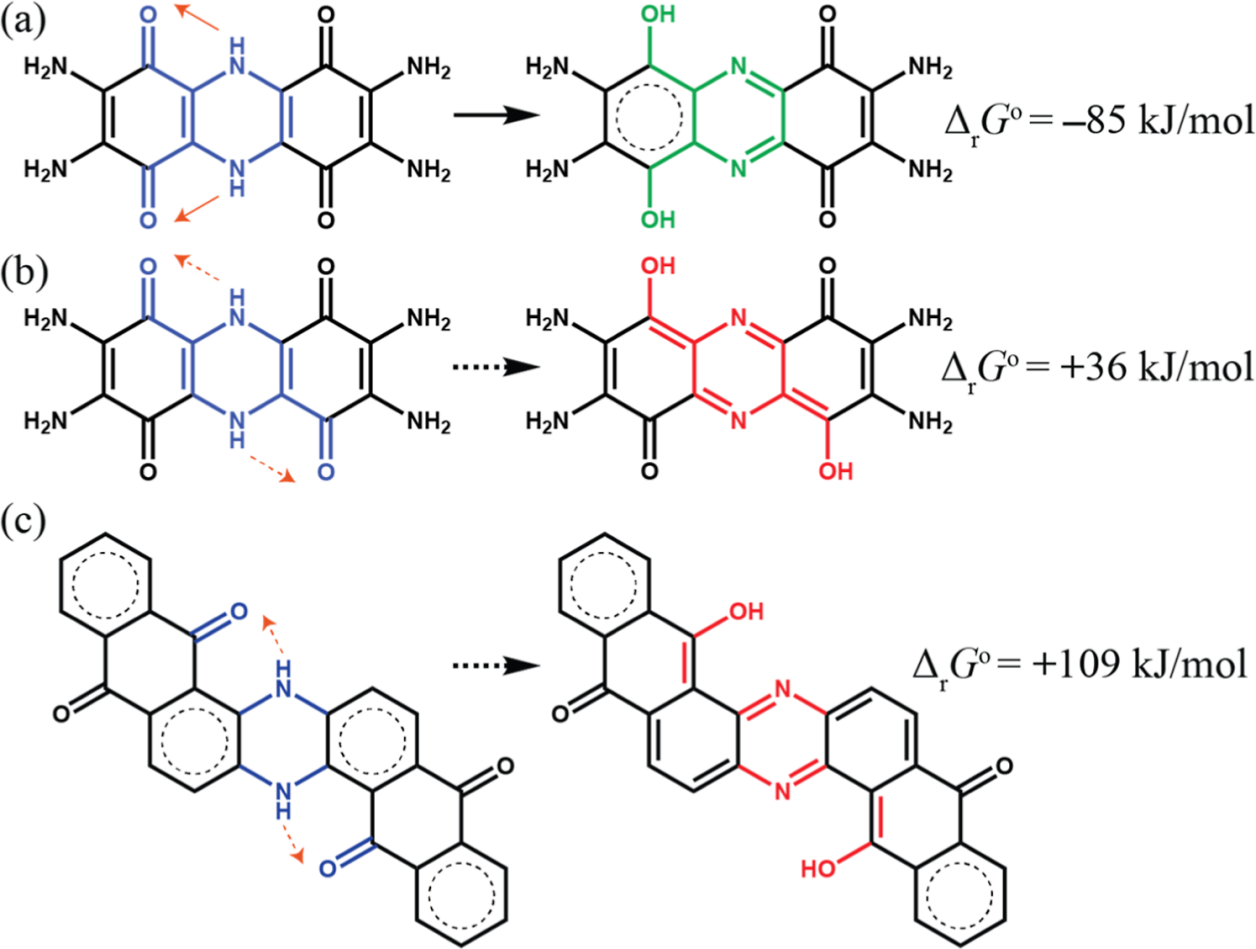
Relative Instability of TAQ According to Quantum-Chemical Calculations of Δ_r_ *G*°^a^

a (a) Thermodynamically favorable conversion of TAQ into a hydroquinone form of TAPT, which forms an aromatic ring (mobile sextet of π-electrons, indicated by a dashed circle). (b,c) Two thermodynamically forbidden processes: (b) unfavorable tautomerization of TAQ and (c) hypothetical, unfavorable tautomerization of indanthrone.

### ^15^N Intensity near 105 ppm in “TAQ” and TAPT

Broad intensity is observed near 105 ppm in the spectrum of ^15^N_6_-TAQ. While its assignment is challenging, this band is not a distinguishing feature of “TAQ” because it is not observed in “TAQ” without ^15^N enrichment (Figure 3c), Furthermore, this broad spectral feature is also observed in TAPT (Figure 3a). Consequently, this minor spectral feature has no bearing on our conclusion that “TAQ” is TAPT.

Dipolar dephasing shows that this is signal of N bonded to H. The ^15^N relaxation of this signal is not faster than that of the NH_2_ groups. The ^1^H chemical shift is around 8.5 ppm. This might be the signal of NH_2_ ortho to NH_3_^+^, as in Figure 8b, although the characteristic ^13^C NMR peak near 100 ppm is not observed. The same issue of absence of characteristic ^13^C signals at <125 ppm (see the predicted chemical shifts in Figure 1b,c) makes assignment to a small fraction of TAQ molecules unlikely. Maybe these are signals of the complicated polycondensation products that have been reported.^16,20,21^

### Dihydrophenazine in Indanthrone

Indanthrone, see Scheme 3c, contains a stable dihydrophenazine core. Why this structural unit is stable in indanthrone but not in TAQ can be explained on the basis of Scheme 3: Tautomerization with hydrogen transfer from N to O is energetically unfavorable, see Scheme 3c. Four independent sextets of π-electrons likely provide significant aromatic stabilization of indanthrone, and NICS scans, see Figure S13, reveal that its central dihydropyrazine core is less antiaromatic than in TAQ. There is also a complicated change in bonding, for instance with weaker C–N π-bonds formed at the expense of slightly stronger C–O π-bonds. A similar process in TAQ, see Scheme 3b, would also be moderately unfavorable. The favorable tautomeric conversion of TAQ in Scheme 3a has no analogue in indanthrone due to indanthrone’s lower symmetry.

### Implications

A reviewer of this study inquired about the “practical significance of clarifying these organic cathode structures.” In the long run, materials design is hampered if the actual structure of the materials under study is not accurately known. We have shown that the previously proposed structure of “TAQ” has distinctly different properties than its actual TAPT structure, such as a different bandgap, an antiaromatic central ring, and a different number of lithium binding sites, as pointed out in the caption of Scheme 1. Carefully considered, the reviewer’s question is moot because TAQ has never been made and probably cannot be made, according to our energetic analyses. It has been proposed^21^ that “the dihydropyrazine linkage [of TAQ] is crucial for enabling significant electronic delocalization between the two neighboring diaminobenzoquinone moieties.” And others^16^ opined that EPR “demonstrates the formation of cationic radicals on the nitrogen of the middle piperazine ring” of “TAQ” (designated as TDT in that study). These beautiful explanations are all meaningless because they are based on the structure that we have disproved—there is no “dihydropyrazine linkage” and no “piperazine ring” in “TAQ”, and it probably cannot be made.

As a result of our findings, at least two studies of “TAQ” in high-impact journals in 2023 and 2024 ^16,20,21^ have lost most of their claimed novelty because the authors actually just repeated measurements of electrochemical and other properties of TAPT that had already been published in 2022.^9^ In other words, most data in these publications from 2023 and 2024 can be found in the earlier paper from 2022 ^9^ or are based on structural assumptions that we have shown to be incorrect. Maybe our study can be a wake-up call to the organic-materials community to require more reliable structural characterization of insoluble materials, ideally by advanced solid-state NMR as demonstrated here, and to develop a mechanism to deal with published studies that have turned out to have little novelty because they were built on incorrect structural claims and were, in effect, duplicative. Furthermore, the rejection of this study by *JACS*, approved at the highest level, has revealed that the Executive Editor of *JACS* and his Editorial Board do not feel an obligation to correct errors^22^ published in *JACS*, despite a recent analysis^60^ highlighting the importance of truth and validation of facts in chemistry.

While some materials chemists may be focused more on exceeding performance benchmarks than on validating structure, and initial reviewers of this work expressed only limited interest in the difference between the claimed and actual chemical structure of “TAQ”, the patent system recognizes “composition of matter” as a central concept. Our study shows that the composition of “TAQ” is different than claimed in the literature and may call into question intellectual property^35,37^ built on incorrect interpretation of distorted ssNMR spectra^14,16,20,21^ recorded with the inadequate conventional cross-polarization method.

## Conclusions

Our comprehensive solid-state NMR study has rigorously elucidated the chemical structure of TAPT and “TAQ” organic electrode materials. They show essentially identical ^13^C and ^15^N chemical shifts; differences in line broadening can be attributed to differences in the concentration of unpaired electron spins, which is confirmed by major differences in ^1^H and ^13^C spin–lattice relaxation. The width of the peaks of ^15^N_6_-“TAQ” is intermediate between those in “TAQ” and TAPT, indicating a continuum of physical properties for the same underlying chemical structure. The observation of two ^15^NH_2_ and two ^13^C–NH_2_ signals is shared with TABQ, a highly symmetric model compound, where it is traced to asymmetric hydrogen bonding in the solid state. Slow ^13^C­{^1^H} and ^15^N­{^1^H} dipolar dephasing demonstrates that the pyrazine ring of both TAPT and “TAQ” is free of any hydrogen, which avoids destabilizing antiaromaticity. The characteristic nonprotonated 309 ppm pyrazine nitrogen of TAPT is quantitatively observed in direct-polarization ^15^N NMR of ^15^N_6_-“TAQ” with and without ^1^H decoupling. 2D NMR confirms that the spectrally resolved amines in “TAQ” are *ortho* to each other. The MALDI-TOF mass spectra of TAPT and “TAQ” also agree. All these observations lead to the conclusion that “TAQ” is TAPT, whose NMR and mass spectra agree with expectations. Quantum-chemical stability analysis has shown that formation of TAQ is not thermodynamically favorable and that TAQ is not stable, being able to spontaneously tautomerize into a reduced form of TAPT. These findings imply that publications of the electrochemical materials properties of “TAQ” were duplicative, preempted by earlier analyses of TAPT, and that the composition of “TAQ” reported in a patent application is incorrect.

## Supplementary Material



## Notes

Synthesis of ^15^N_4_-TABQ; NMR pulse sequences used; NICS aromaticity scans of TAPT, TAQ, and indanthrone; additional ^13^C NMR spectra and relaxation data of “TAQ”; analysis of relaxation by randomly distributed unpaired electrons; CODEX data of ^15^N_6_-“TAQ”; and additional NMR spectra of indanthrone (PDF)

## References

[1] Blomgren G. E. (2017). The Development
and Future of Lithium Ion Batteries. J. Electrochem.
Soc..

[2] Liu C., Neale Z. G., Cao G. (2016). Understanding Electrochemical Potentials
of Cathode Materials in Rechargeable Batteries. Mater. Today.

[3] Finkelstein S. H., Ricci M., Bötticher T., Schmidt-Rohr K. (2024). How Lithium-ion
Batteries Work Conceptually: Thermodynamics of Li Bonding in Idealized
Electrodes. Phys. Chem. Chem. Phys..

[4] Mao M., Luo C., Pollard T. P., Hou S., Gao T., Fan X., Cui C., Yue J., Tong Y., Yang G. (2019). A Pyrazine-based
Polymer for Fast-charge Batteries. Angew. Chem.,
Int. Ed..

[5] Jerng S. E., Chang B., Shin H., Kim H., Lee T., Char K., Choi J. W. (2020). Pyrazine-linked
2D Covalent Organic
Frameworks as Coating Material for High-nickel Layered Oxide Cathodes
in Lithium-ion Batteries. ACS Appl. Mater. Interfaces.

[6] Menart S., Luzanin O., Pirnat K., Pahovnik D., Mos̆kon J. e., Dominko R. (2024). Design of Organic Cathode Material
Based on Quinone
and Pyrazine Motifs for Rechargeable Lithium and Zinc Batteries. ACS Appl. Mater. Interfaces.

[7] Gao Y., Li G., Wang F., Chu J., Yu P., Wang B., Zhan H., Song Z. (2021). A High-performance Aqueous Rechargeable
Zinc Battery Based on Organic Cathode Integrating Quinone and Pyrazine. Energy Storage Mater..

[8] Yang M., Hu W., Li J., Chen T., Zhao S., Chen X. a., Wang S., Jin H. (2024). Long Cycle Life for Rechargeable
Lithium Battery Using Organic Small Molecule Dihydrodibenzo [c, h]­[2,
6] naphthyridine-5, 11-dione as a Cathode after Isoindigo Pigment
Isomerization. Adv. Sci..

[9] Li Z., Jia Q., Chen Y., Fan K., Zhang C., Zhang G., Xu M., Mao M., Ma J., Hu W. (2022). A Small
Molecular Symmetric All-organic Lithium-ion Battery. Angew. Chem., Int. Ed..

[10] Fan K., Li J., Xu Y., Fu C., Chen Y., Zhang C., Zhang G., Ma J., Zhai T., Wang C. (2023). Single Crystals
of a Highly Conductive Three-Dimensional Conjugated Coordination Polymer. J. Am. Chem. Soc..

[11] Wang Y., Wang J., Peng J., Jiang Y., Zhu Y., Yang Y. (2024). Crafting Core-Shell Heterostructures with Enriched Active Centers
for High-Energy-Density Symmetric Lithium-Ion Batteries. ACS Nano.

[12] Zhang C., Li Z., Guan L., Fu M., Fan K., Chen Y., Zhang G., Zou J., Wang C. (2023). Anti-aggregation Triggering
Molecular Transformation and Boosting Stable Sodium Storage. Cell Rep. Phys. Sci..

[13] Wu Y., Ye H., Li Y. (2025). Molecular
Engineering of Organic Electrode Materials
for Beyond Lithium-Ion Batteries. Adv. Funct.
Mater..

[14] Yao Y., Pei M., Su C., Jin X., Qu Y., Song Z., Jiang W., Jian X., Hu F. (2024). A Small-Molecule
Organic
Cathode with Extended Conjugation toward Enhancing Na^+^ Migration
Kinetics for Advanced Sodium-Ion Batteries. Small.

[15] Ji W., Du D., Liang J., Li G., Feng G., Yin Z., Zhou J., Zhao J., Shen Y., Huang H. (2023). Aqueous Zn-organic Batteries: Electrochemistry and Design Strategies. Battery Energy.

[16] Lin L., Lin Z., Zhu J., Wang K., Wu W., Qiu T., Sun X. (2023). A Semi-conductive
Organic Cathode Material Enabled by Extended Conjugation
for Rechargeable Aqueous Zinc Batteries. Energy
Environ. Sci..

[17] Qi Y., Zhao H., Chen L., Lei Y. (2026). Solubility Challenges
and Strategies for Organic Sodium-Ion Batteries: Status and Perspectives. Small.

[18] Wang Y., Zhao Y., Xu X., Gao W., Zhang Q., Huang W. (2024). A Review on the Role of Hydrogen
Bonds in Organic Electrode Materials. Batter.
Supercaps.

[19] Lakraychi A. E., Yao Y. (2023). Designing Organic Pseudocapacitors
through Molecular Hybridization. Joule.

[20] Chen T., Banda H., Yang L., Li J., Zhang Y., Parenti R., Dincă M. (2023). High-rate,
High-capacity Electrochemical
Energy Storage in Hydrogen-bonded Fused Aromatics. Joule.

[21] Chen T., Banda H., Wang J., Oppenheim J. J., Franceschi A., Dincǎ M. (2024). A Layered
Organic Cathode for High-energy,
Fast-charging, and Long-Lasting Li-ion Batteries. ACS Cent. Sci..

[22] Chen T., Wang J., Tan B., Zhang K. J., Banda H., Zhang Y., Kim D.-H., Dincǎ M. (2025). High-energy,
High-power Sodium-ion Batteries from a Layered Organic Cathode. J. Am. Chem. Soc..

[23] Han X., Yi F., Sun T., Sun J. (2012). Synthesis and Electrochemical Performance
of Li and Ni 1, 4, 5, 8-Naphthalenetetracarboxylates as Anodes for
Li-ion Batteries. Electrochem. Commun..

[24] Kolek M., Otteny F., Schmidt P., Mück-Lichtenfeld C., Einholz C., Becking J., Schleicher E., Winter M., Bieker P., Esser B. (2017). Ultra-high
Cycling
Stability of Poly (vinylphenothiazine) as a Battery Cathode Material
Resulting from π-π Interactions. Energy Environ. Sci..

[25] Liang Y., Zhang P., Chen J. (2013). Function-oriented
Design of Conjugated
Carbonyl Compound Electrodes for High Energy Lithium Batteries. Chem. Sci..

[26] Luo Z., Liu L., Zhao Q., Li F., Chen J. (2017). An Insoluble Benzoquinone-based
Organic Cathode for Use in Rechargeable Lithium-ion Batteries. Angew. Chem., Int. Ed..

[27] Renault S., Oltean V. A., Araujo C. M., Grigoriev A., Edström K., Brandell D. (2016). Superlithiation of Organic Electrode
Materials: the Case of Dilithium Benzenedipropiolate. Chem. Mater..

[28] Schon T. B., McAllister B. T., Li P.-F., Seferos D. S. (2016). The Rise of Organic
Electrode Materials for Energy Storage. Chem.
Soc. Rev..

[29] Shea J. J., Luo C. (2020). Organic Electrode Materials
for Metal Ion Batteries. ACS Appl. Mater. Interfaces.

[30] Song Z., Qian Y., Liu X., Zhang T., Zhu Y., Yu H., Otani M., Zhou H. (2014). A Quinone-based Oligomeric
Lithium
Salt for Superior Li-organic Batteries. Energy
Environ. Sci..

[31] Song Z., Zhou H. (2013). Towards Sustainable
and Versatile Energy Storage Devices: An Overview
of Organic Electrode Materials. Energy Environ.
Sci..

[32] Wang S., Wang L., Zhang K., Zhu Z., Tao Z., Chen J. (2013). Organic Li_4_C_8_H_2_O_6_ Nanosheets
for Lithium-ion Batteries. Nano Lett..

[33] Patel, P. Metal-free Cathode Rivals Commercial Battery Performance. *Chemical & Engineering News* . 2024, https://cen.acs.org/energy/energy-storage-/Metal-free-cathode-rivals-commercial-battery-performance/102/web/2024/01 (accessed Febuary 16, 2024).

[34] Wang, C. ; Li, Z. ; Chen, Y. ; Zhang, C. Organic Electrode Material Universal for Alkali Metal Ion Battery and Application thereof. CN 114204020 A, 2022.

[35] Dinca, M. ; Banda, H. ; Chen, T. Fused Aromatic Molecules as Electrode Materials. WO 2023022750 A1, 2024.

[36] Richard, I. Lamborghini Licenses MIT’s New TAQ Battery—Fast Charging, Organic, and MORE Features. *Tech Times* . 2024, https://www.techtimes.com/articles/300850/20240121/lamborghini-licenses-mit-s-new-taq-batteryfast-charging-organic-more.htm (accessed April 24, 2025).

[37] Scott, A. On our Radar 2025. *Chemical & Engineering News* . 2025, https://cen.acs.org/business/start-ups/on-our-radar-2025/103/web/2025/11 (accessed Febuary 14, 2026).

[38] Kaim W. (1983). The Versatile
Chemistry of 1,4-Diazines: Organic, Inorganic and Biochemical Aspects. Angew. Chem., Int. Ed. Engl..

[39] Schmidt-Rohr K. (2015). Why Combustions
are Always Exothermic, Yielding about 418 kJ per Mole of O2. J. Chem. Educ..

[40] Yuan S., Duan P., Berthier D. L., León G., Sommer H., Saint-Laumer J. Y. d., Schmidt-Rohr K. (2020). Multinuclear
Solid-state NMR of Complex Nitrogen-rich Polymeric Microcapsules:
Weight Fractions, Spectral Editing, Component Mixing, and Persistent
Radicals. Solid State Nucl. Magn. Reson..

[41] Fang X., Mao J., Levin E., Schmidt-Rohr K. (2009). Nonaromatic
Core-shell Structure
of Nanodiamond from Solid-state NMR Spectroscopy. J. Am. Chem. Soc..

[42] Pagenkopf B. (2005). ACD/HNMR Predictor
and ACD/CNMR Predictor Advanced Chemistry Development, Inc. (ACD/Labs). J. Am. Chem. Soc..

[43] Frisch, M. J. ; Trucks, G. W. ; Schlegel, H. B. ; Scuseria, G. E. ; Robb, M. A. ; Cheeseman, J. R. ; Scalmani, G. ; Barone, V. ; Petersson, G. A. ; Nakatsuji, H. ; Gaussian 16, Rev. B.02; Gaussian, Inc.: Wallingford, CT, 2016.

[44] Schaefer J., Stejskal E. O. (1976). Carbon-13 nuclear
magnetic resonance of polymers spinning
at the magic angle. J. Am. Chem. Soc..

[45] Fung B. M., Khitrin A., Ermolaev K. (2000). An Improved Broadband Decoupling
Sequence for Liquid Crystals and Solids. J.
Magn. Reson..

[46] Mao J.-D., Schmidt-Rohr K. (2003). Recoupled
Long-range C-H Dipolar Dephasing in Solid-state
NMR, and its Use for Spectral Selection of Fused Aromatic Rings. J. Magn. Reson..

[47] Dixon W., Schaefer J., Sefcik M., Stejskal E., McKay R. (1982). Total Suppression
of Sidebands in CPMAS C-13 NMR. J. Magn. Reson..

[48] Sato M., Takeda T., Hoshino N., Akutagawa T. (2017). Electronic
and Crystal Structures of 1,2,3-Triazole-fused p-Benzoquinone Derivatives. CrystEngComm.

[49] Lodewyk M. W., Siebert M. R., Tantillo D. J. (2012). Computational
Prediction of ^1^H and ^13^C Chemical Shifts: A
Useful Tool for Natural
Product, Mechanistic, and Synthetic Organic Chemistry. Chem. Rev..

[50] Marenich A. V., Cramer C. J., Truhlar D. G. (2009). Universal
Solvation Model Based on
Solute Electron Density and on a Continuum Model of the Solvent Defined
by the Bulk Dielectric Constant and Atomic Surface Tensions. J. Phys. Chem. B.

[51] Gershoni-Poranne R., Stanger A. (2014). The NICS-XY-Scan: Identification of Local and Global
Ring Currents in Multi-Ring Systems. Chem.—Eur.
J..

[52] Johnson R. L., Schmidt-Rohr K. (2014). Quantitative
Solid-state ^13^C NMR with Signal
Enhancement by Multiple Cross Polarization. J. Magn. Reson..

[53] Blumberg W. E. (1960). Nuclear
Spin-Lattice Relaxation Caused by Paramagnetic Impurities. Phys. Rev..

[54] Furman G. B., Kunoff E. M., Goren S. D., Pasquier V., Tinet D. (1995). Nuclear Spin-lattice
Relaxation via Paramagnetic Impurities in Solids with Arbitrary Space
Dimension. Phys. Rev. B: Condens. Matter Mater.
Phys..

[55] deAzevedo E. R., Hu W.-G., Bonagamba T. J., Schmidt-Rohr K. (2000). Principles
of Centerband-only Detection of Exchange in Solid-state Nuclear Magnetic
Resonance, and Extension to Four-time Centerband-only Detection of
Exchange. J. Chem. Phys..

[56] Barrow, M. Mass Calculations: Mass Error and m/z from Formula, Barrow Group, Department of Chemistry, University of Warwick, 2021. https://warwick.ac.uk/fac/sci/chemistry/research/barrow/barrowgroup/calculators/mass_errors/ (accessed January 16, 2025).

[57] Lou X., Sinkeldam R. W., van Houts W., Nicolas Y., Janssen P. G. A., van
Dongen J. L. J., Vekemans J. A. J. M., Meijer E. W. (2007). Double Cation Adduction
in Matrix-assisted Laser Desorption/ionization
Time-of-flight Mass Spectrometry of Electron Deficient Anthraquinone
Derivatives. J. Mass Spectrom..

[58] Napolitano M. P., Kuo P.-C., Johnson J. V., Arslanoglu J., Yost R. A. (2017). Tandem Mass Spectrometry of Laser-Reduced Anthraquinones
for Painted Works and Dyed Cultural Artifacts. Int. J. Mass Spectrom..

[59] Sugawara T., Takasu I. (1999). Tautomerism in the
Solid State. Adv. Phys. Org. Chem..

[60] Oldach L. (2026). Editorial:
Facts are in crisis. What are we going to do?. C&EN Global Enterprise.

