# Supplementary material for: Comparison of the Structure of Advanced Organic Electrode Materials, TAPT and “TAQ”, by Multinuclear NMR and Stability Analysis
Source: J Phys Chem A. 2026 Jul 17;130(30):5878–90. doi: 10.1021/acs.jpca.6c03213 (PMC13430626; doi:10.1021/acs.jpca.6c03213)
Supplement: Supplementary file 1 [file jp6c03213_si_001.pdf]

Supporting Information

for

Comparison of the Structure of Advanced Organic Electrode Materials, TAPT and "TAQ", by Multinuclear NMR and Stability Analysis

J. Litterio[1a], S. H. Finkelstein[1a], Z. Sun[1a], Kayla Cerri[1], K. Schmidt-Rohr[1*]
[1]: Department of Chemistry, Brandeis University, Waltham, MA, USA.

Table of Contents

Synthesis of $^{15}N_4$-TABQ……………………………………………………………p. S1
Scheme S1. Synthesis of $^{15}N_4$-TABQ……………………………………………p. S2
Scheme S2. NMR pulse sequences used in this work………………………………..p. S3
Figure S1. $NICS_{zz}$ aromaticity scans of TAQ and TAPT…………………………..p. S4
Figure S2. Series of direct-polarization ^{13}C NMR spectra…………………………p. S5
Figure S3. Comparison of multiCP and direct-polarization ^{13}C NMR……………p. S6
Figure S4. Peak intensities in saturation recovery……………………………………p. S7
Nuclear spin-lattice relaxation by randomly distributed unpaired electrons….p. S7
Figure S5. Direct polarization ^{15}N NMR at 7 kHz MAS…………………………...p. S10
Figure S6. Series of multiCP ^{13}C NMR spectra……………………………………p. S10
Figure S7. ^{13}C NMR of a model compound after long-range $^{13}C\{^1H\}$ dephasing…p. S11
Figure S8. ^{15}N exchange NMR in $^{15}N_6$-"TAQ" at 4 kHz MAS……………………p. S12
Figure S9. MAS and static ^{13}C spectra of TABQ and TABQ·2HCl………………..p. S13
Figure S10. ^1H-^{13}C HetCor spectra of TABQ and "TAQ"…………………………p. S14
Figure S11. MultiCP and ^1H-^{13}C HetCor spectra of indanthrone…………………...p. S15
Figure S12. ^{13}C chemical shift predictions for tautomers of TAQ…………………p. S16
Figure S13. NICS aromaticity scans of TAQ, TAPT, and indanthrone……………..p. S17

Synthesis of $^{15}N_4$-TABQ. Reactions were carried out under normal atmospheric conditions. All reactions were stirred and heated using a Heidolph Hei-Plate Mix 'n' Heat Core with a heating mantle. Reagents were used without further purification. Intermediate products in the synthesis

scheme were characterized using a Bruker Avance Neo 400 solution-NMR spectrometer and a Thermo Scientific Nicolet iS5 FT-IR spectrometer.

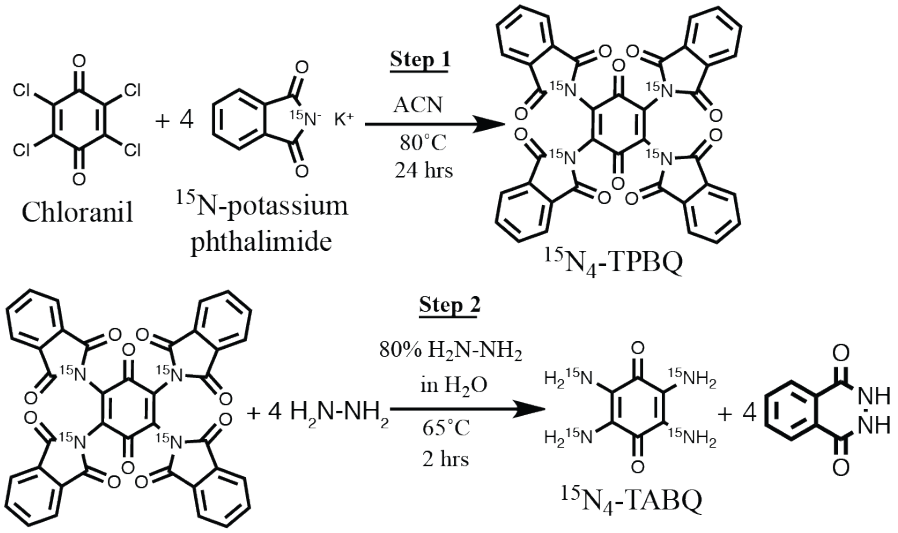

Scheme S1. Synthesis of $^{15}N_4$-TABQ, adapted from previous literature.[1, 2]

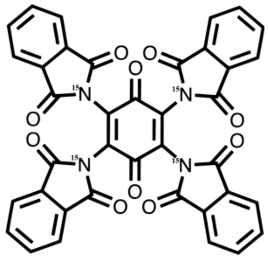

$^{15}N_4$-tetra-phthalimido-*p*-benzoquinone ($^{15}N_4$-TPBQ) 2. $^{15}N_4$-potassium phthalimide from Cambridge Isotope Labs (Lot #: PR-33228)[3] (15.07 g, 81.4 mmol) was added to a suspension of chloranil (4.99 g, 20.3 mmol) in acetonitrile (50.0 mL) and refluxed for 24 hours. The reaction mixture was filtered and washed successively with DMF and cold water until the filtrate ran clear. The gray/brown solid was dried in an oven overnight at 100 °C, before a gray solid was isolated. (5.83 g, 8.5 mmol, 41.6% yield).

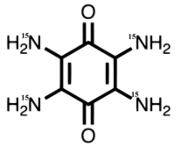

$^{15}N_4$-2,3,5,6-tetraaminobenzoquinone ($^{15}N_4$-TABQ) 3. Hydrazine hydrate (80 wt% hydrazine; 10 mL) was slowly added to the reaction flask charged with $^{15}N_4$-TPBQ (2.75 g, 3.9 mmol) and set to react in an oil bath at 65 °C, open to atmosphere, for two hours. The reaction was cooled to room temperature and placed in an ice bath before it was filtered and washed with cold DI water until the filtrate ran clear. The isolated product was put in an oven at 100 °C overnight and deep-blue/black crystals were obtained. (0.46 g, 2.7 mmol, 69% yield).

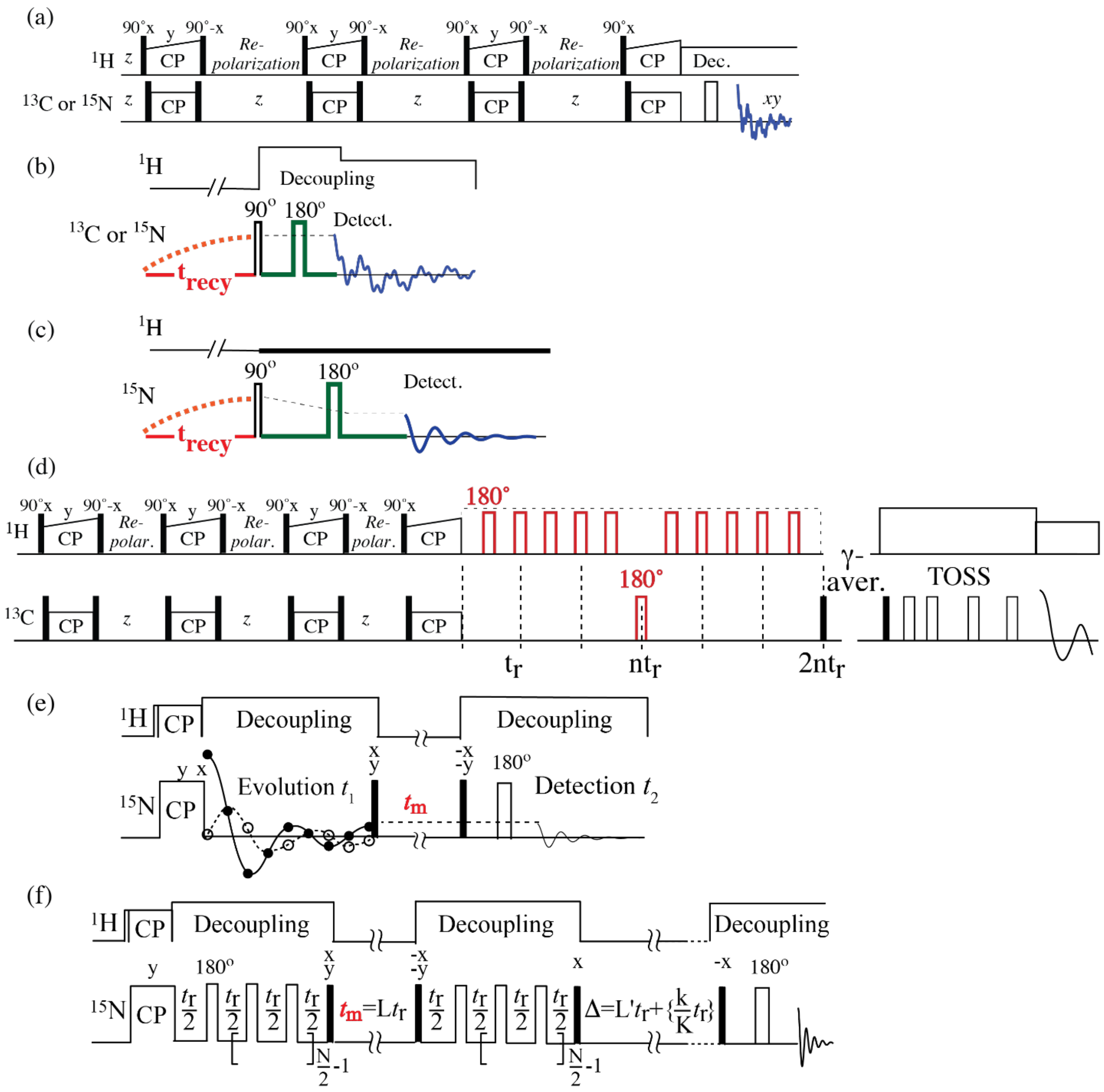

Scheme S2. NMR pulse sequences used in this work. Note the rotation-synchronized Hahn spin echo with EXORCYCLEd[4] π-pulse (or TOSS) before detection in all experiments.

(a) ^{1}H-^{13}C multiple-cross polarization (multiCP) [5] NMR (in Figs. 3, 6, 8, S3, S6, S11, S12).

(b) Direct-polarization or saturation-recovery ^{13}C/^{15}N NMR (in Figs. 1, 2, 4, S2, S4, S5).

(c) Direct-polarization ^{15}N NMR without ^{1}H decoupling (in Fig. 4 and S5).

(d) ^{1}H-^{13}C long-range recoupled dipolar dephasing NMR at 7 kHz MAS, with a 180° pulse every half rotation period $t_r/2$, before a z-period with γ-averaging[6]. Total suppression of spinning-sidebands (TOSS)[7] is applied for 2 t_r before detection (in Figs. 5, S7). The S_0 reference spectrum is obtained with full ^{1}H decoupling (dashed rectangle) instead of the recoupling pulses.

(e) ^{1}H-^{15}N cross polarized 2D exchange ^{15}N NMR (in Fig. 7).

(f) CODEX ^{15}N NMR at 4 kHz MAS (in Fig. S8).

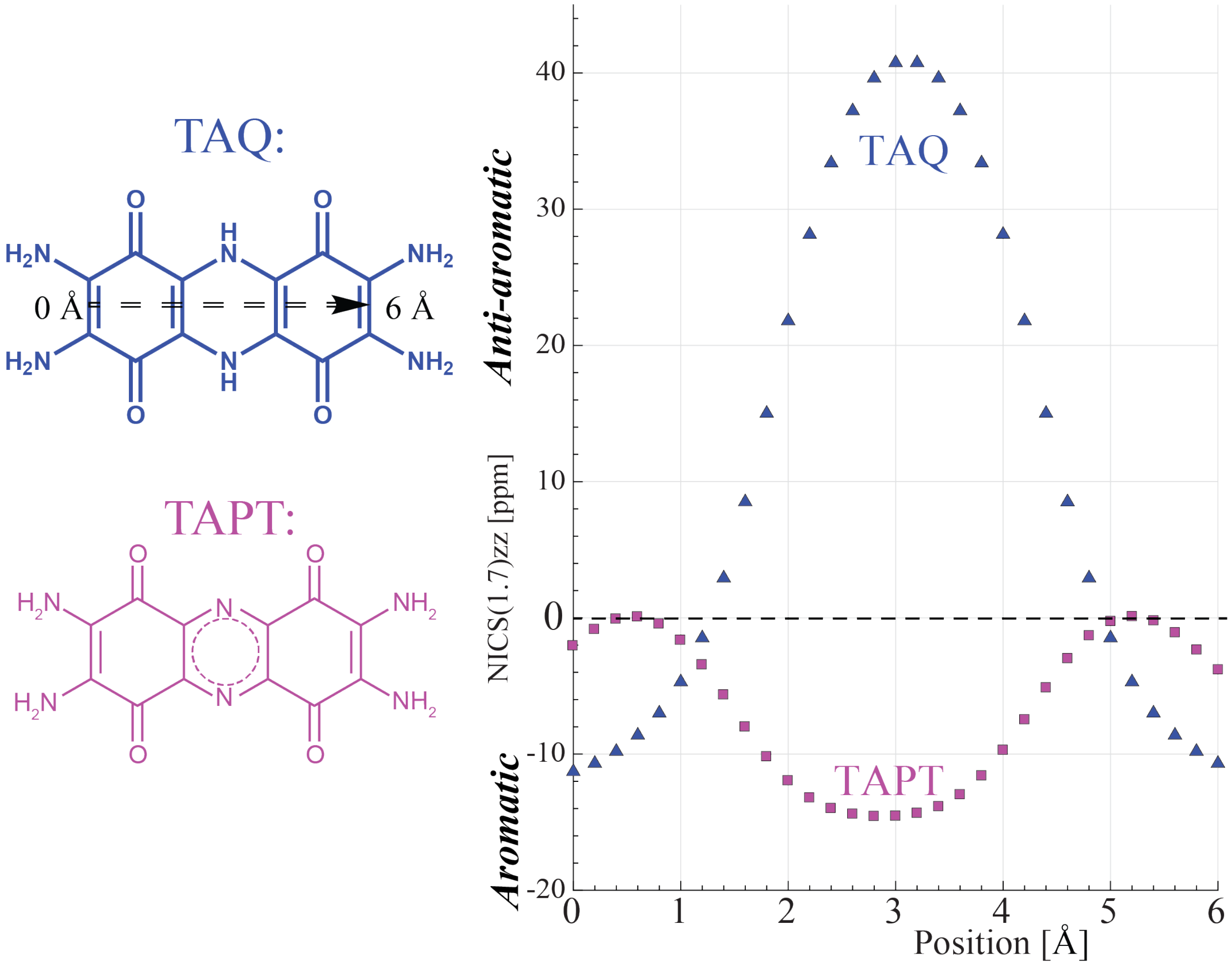

Figure S1. Nucleus-independent chemical shift ($NICS_{zz}$) aromaticity scans across TAQ (blue triangles) and TAPT (magenta squares). The molecules differ by two hydrogen atoms attached to the central ring in TAQ, which make the central ring pronouncedly anti-aromatic.

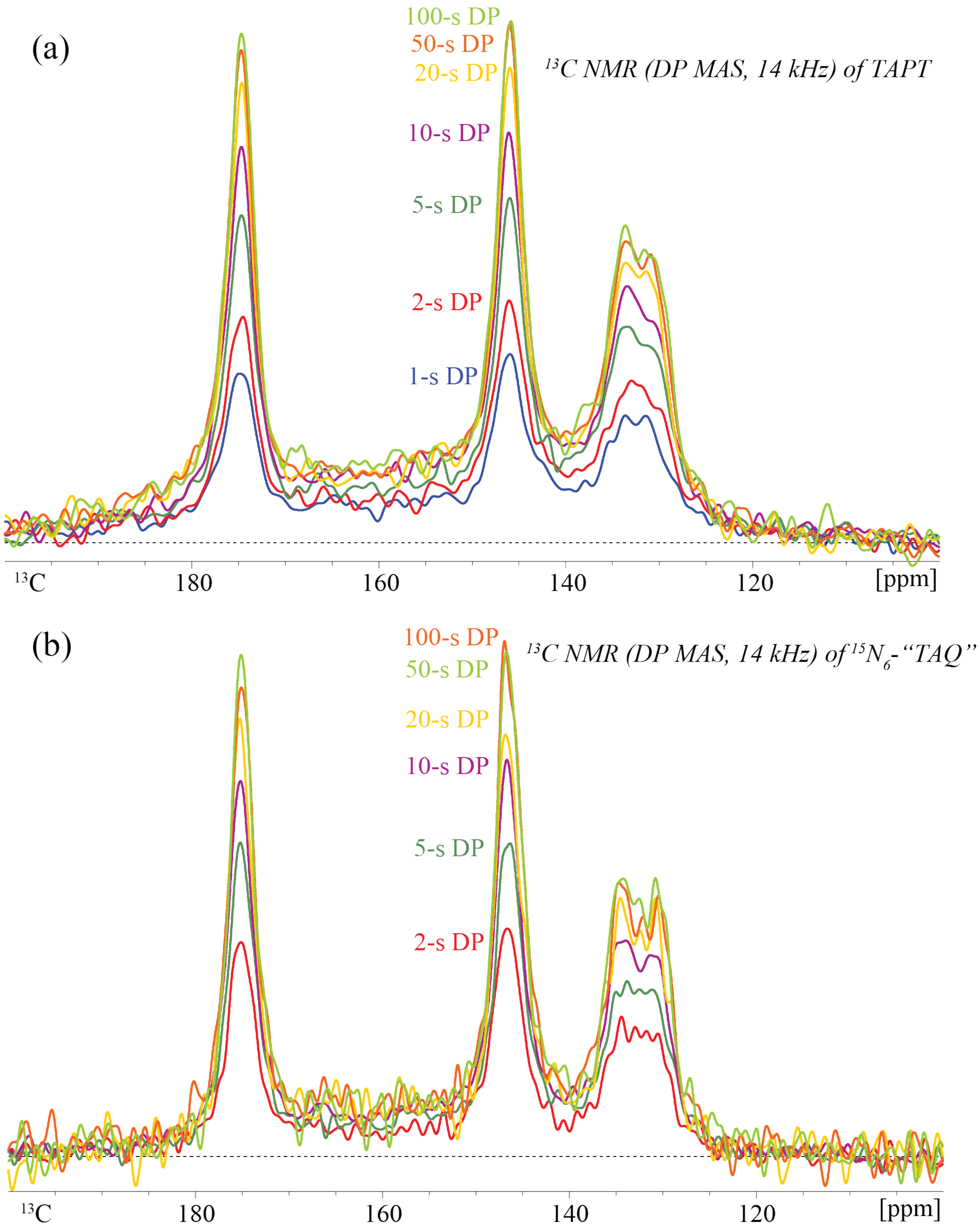

Figure S2. Series of direct-polarization ^{13}C NMR spectra with increasing recycle delay of (a) TAPT and (b) "TAQ". Peak integrals as a function of recycle delay are shown in Figure 2.

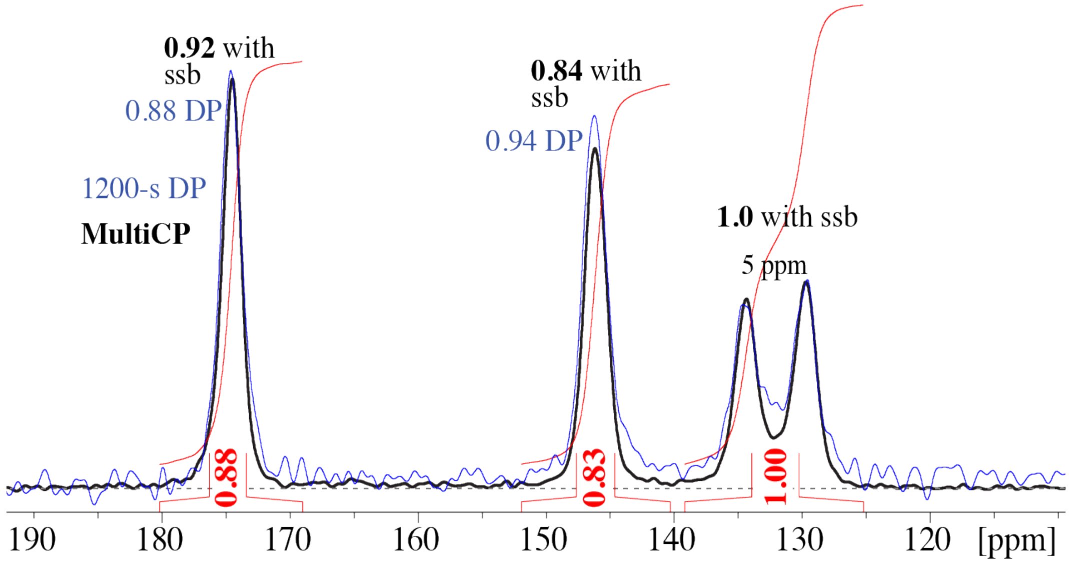

Figure S3. Comparison of multiCP and direct-polarization ^{13}C NMR spectra of "TAQ". Peak integrals are indicated. Thin blue line: DP; thick black line: multiCP.

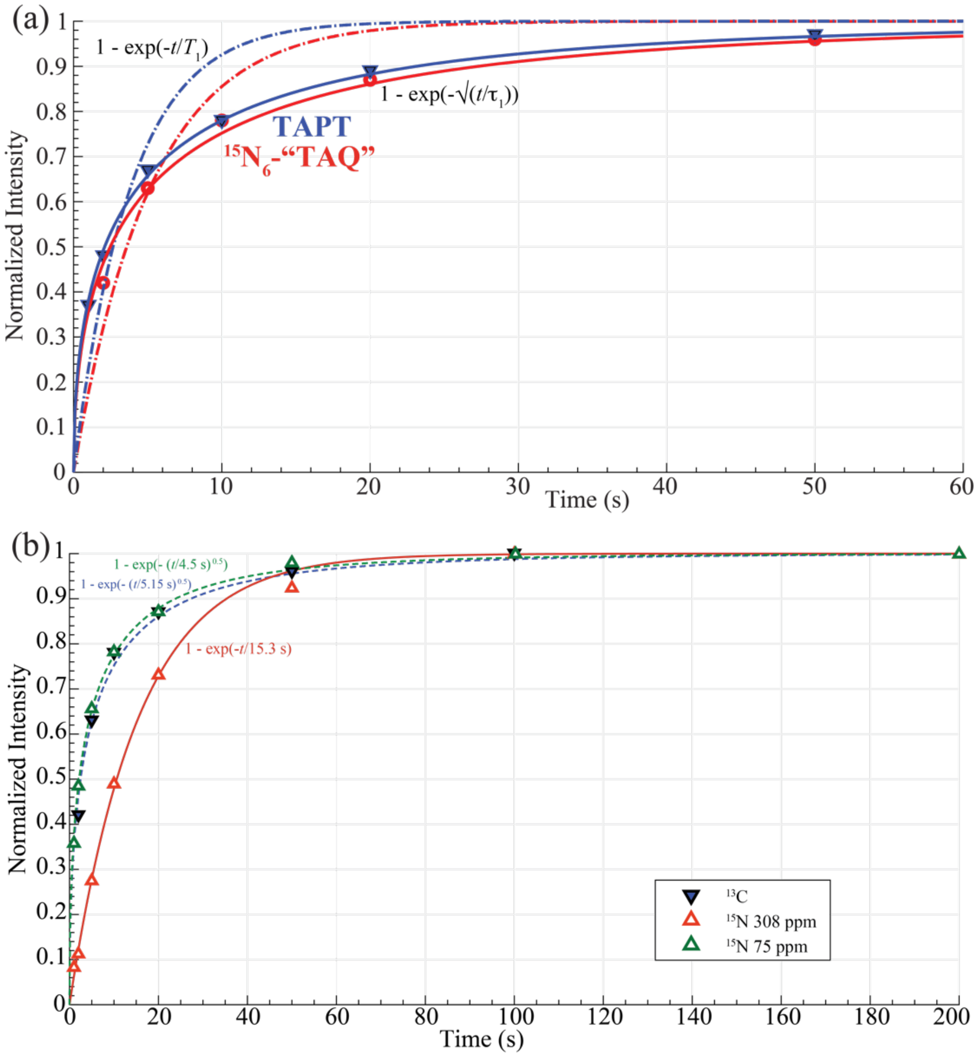

Figure S4. Peak intensities in saturation recovery in (a) ^{13}N NMR of $^{15}N_6$-“TAQ” and TAPT, with good square-root exponential (solid) fits and reviewer-requested poor regular exponential (dash-dotted) fits (expanded relative to Figure 2a). (b) ^{15}N NMR of $^{15}N_6$-“TAQ” with recycle delays between 1 s and 200 s.

Nuclear spin-lattice relaxation by randomly distributed unpaired electrons. In Figure 2a, fast and distinctly nonexponential ^{13}C spin-lattice relaxation is documented. In saturation recovery, the relaxing polarization of nucleus n surrounded by L unpaired electrons at distances r_{nl} is

$$R_n(t) = R_\infty \left(1 - \prod_{l=1}^{L} \exp\left(-\frac{A}{(r_{nl})^6} t\right)\right) \qquad (S1)$$

where A is a constant of the electron-nucleus dipolar coupling, ignoring the weak orientation-dependence for simplicity.[8, 9] This applies to dilute nuclei without spin-diffusion. The observed magnetization is the sum of the contributions from the N nuclei present

$$M(t) = \sum_{n=1}^{N} R_n(t) = N\,\langle R(t)\rangle = N\,R_\infty\,\langle 1 - \prod_{l=1}^{L} \exp\left(-\frac{A}{(r_{nl})^6} t\right)\rangle \qquad (S2)$$

The normalized magnetization is

$$1 - \frac{M(t)}{M(\infty)} = \langle \prod_{l=1}^{L} \exp\left(-\frac{A}{(r_{nl})^6} t\right)\rangle \qquad (S3)$$

This is the expected value of the product of the function $f(r) = \exp(-Ar^{-6}t)$ evaluated at L points that are randomly distributed in volume V with density ρ. The number L of electrons in the volume V follows the Poisson distribution, with $\langle L\rangle = \rho V$. The average can be evaluated using the Poisson probability Prob(L) = $\frac{(\rho V)^L e^{-\rho V}}{L!}$ of having L electrons in the volume V and spatially averaging every $f(r)$ factor:

$$1 - \frac{M(t)}{M(\infty)} = \langle \prod_{l=1}^{L} f(r_l)\rangle = \sum_{L=0}^{\infty} \text{Prob}(L) \frac{1}{V^L} \int_{-\infty}^{\infty} \int_{-\infty}^{\infty} \dots \int_{-\infty}^{\infty} f(r_1) f(r_2) \dots f(r_L)\, d^3r_1 d^3r_2 \dots d^3r_L$$

$$= \sum_{L=0}^{\infty} \frac{(\rho V)^L e^{-\rho V}}{L!} \left(\frac{1}{V} \int_{-\infty}^{\infty} f(r) d^3r\right)^L \qquad (S4)$$

Rearranging terms and recognizing the Taylor series of the exponential function, we obtain

$$1 - \frac{M(t)}{M(\infty)} = \sum_{L=0}^{\infty} \frac{(\rho \int f(r) d^3r)^L}{L!} e^{-\rho V} = \exp\left(\rho \int_{-\infty}^{\infty} f(r) d^3r\right) e^{-\rho V}$$

$$= \exp\left(\rho \int_{-\infty}^{\infty} f(r) d^3r - \rho V\right) = \exp\left(-\rho \int_{-\infty}^{\infty} \big(1 - f(r)\big) d^3r\right) \qquad (S5)$$

The result is known as the Laplace functional of the Poisson point process. Inserting $f(r) = \exp(-Ar^{-6}t)$ recovers equation (11) of Blumberg (1960)[8, 9]:

$$\frac{M(t)}{M(0)} = \exp\left(-4\pi\rho \int_{0}^{\infty} (1 - \exp(-Ar^{-6}t)) r^2 dr\right) \qquad (S6)$$

A change of variable

$$u = Ar^{-6}t, \qquad r = \left(\frac{u}{At}\right)^{-\frac{1}{6}}, \qquad dr = -\frac{(At)^{\frac{1}{6}}}{6}u^{-\frac{7}{6}}du \tag{S7}$$

simplifies the integral to

$$1 - \frac{M(t)}{M(\infty)} = \exp\left(-\frac{2\pi\rho}{3}(At)^{\frac{1}{2}}\int_0^\infty (1 - \exp(-u))u^{-\frac{3}{2}}du\right) \tag{S8}$$

The remaining definite integral is just some number C, so

$$1 - \frac{M(t)}{M(\infty)} = \exp\left(-\left(\frac{2C\pi\rho}{3}A^{\frac{1}{2}}\right)t^{\frac{1}{2}}\right) \tag{S9}$$

The time variable in the exponent has the power of ½ implied by Blumberg and others,[8, 9] which is observed experimentally in Figure 2a.

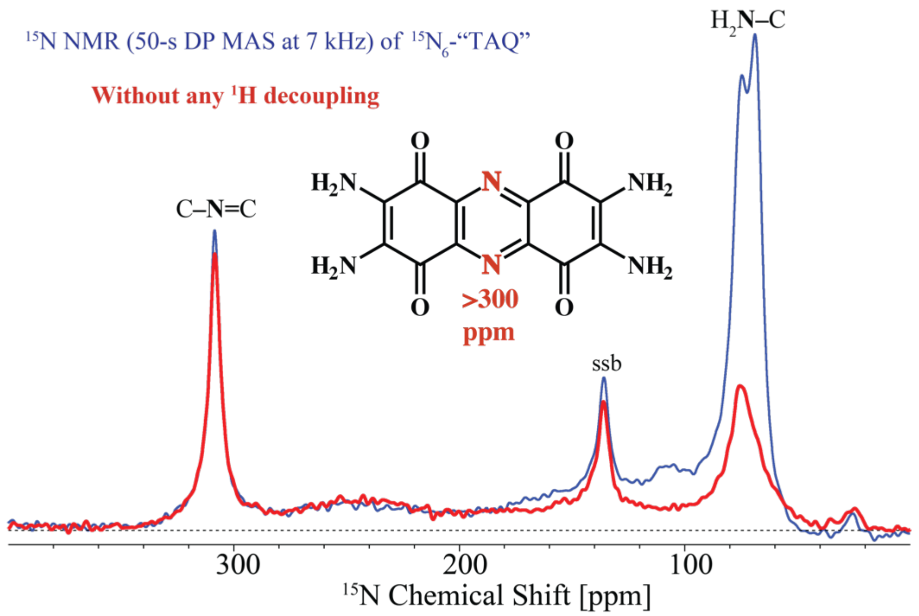

Figure S5. Thin blue line: Nearly fully relaxed direct polarization ^{15}N NMR spectrum of $^{15}N_6$-"TAQ", recorded at 7 kHz MAS with 50-s recycle delay. Thick red line: corresponding spectrum (unscaled) without any ^{1}H decoupling after 1.1 ms of dipolar dephasing and ^{15}N T_2 relaxation. ssb: spinning sideband of the 309-ppm peak (absent from the 14-kHz MAS spectrum in Figure 4).

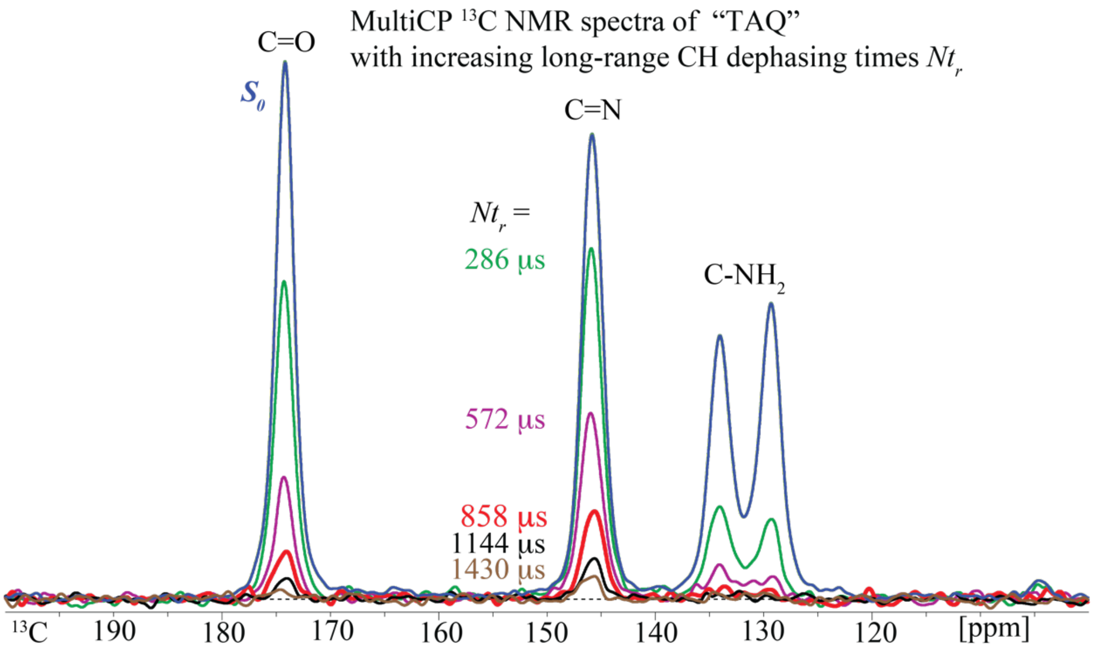

Figure S6. Series of multiCP ^{13}C NMR spectra of "TAQ" after long-range $^{13}C\{^{1}H\}$ dephasing for the indicated times between 0.286 and 1.43 ms. The blue spectrum is the average S_0 spectrum (correcting for T_{2C} relaxation under ^{1}H decoupling).

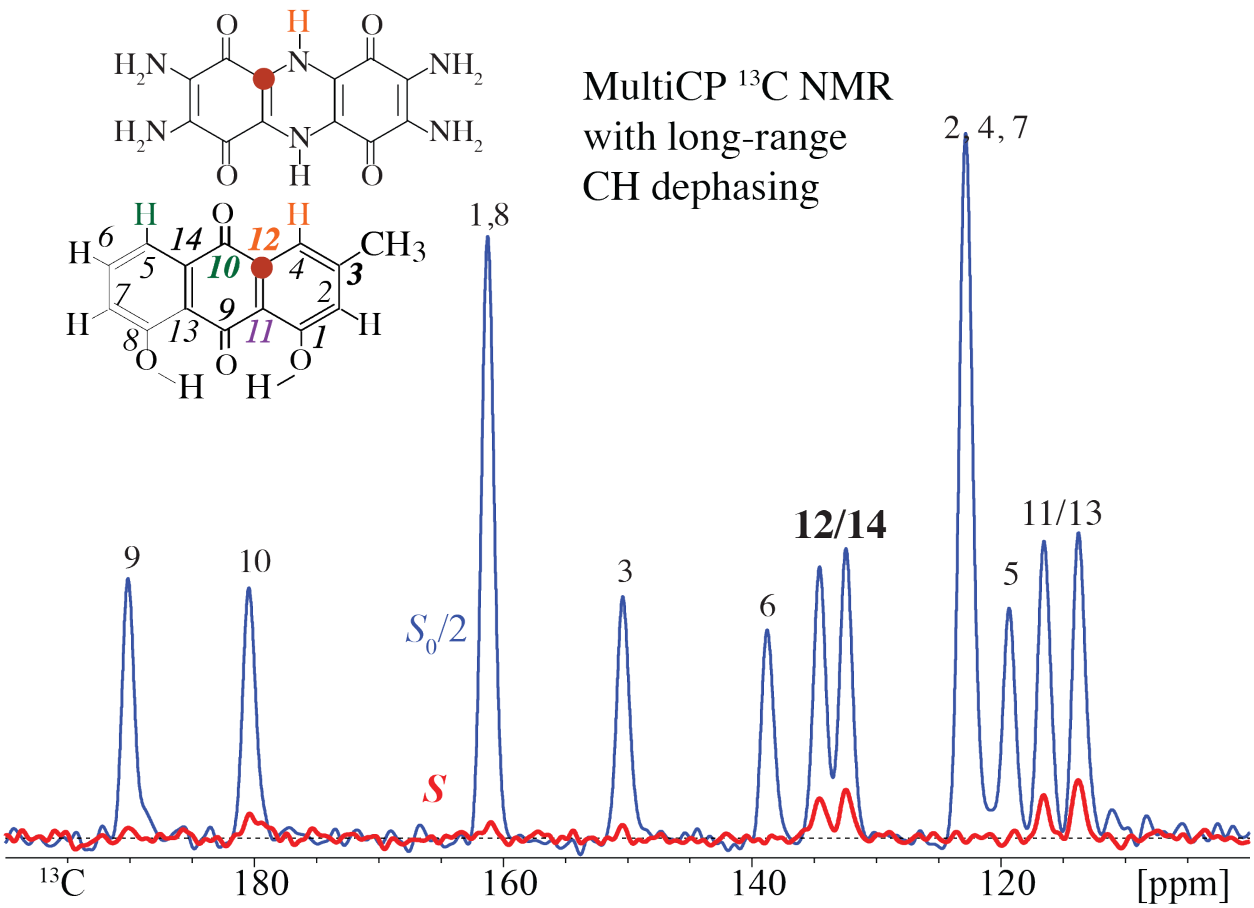

Figure S7. ^{13}C NMR spectrum (in red) of an anthraquinone model compound after long-range ^{13}C{^{1}H} dephasing for 0.858 ms. As in Figure 5, the reference spectrum (in blue) is scaled down by a factor of 0.5 for easier comparability. The inset shows the structure of the model compound in comparison with that of TAQ. Carbon site 12/14 is seen to be analogous to the interior carbon site in TAQ; the spectra show that its dephasing is more pronounced than that observed in "TAQ", see Figure 5.

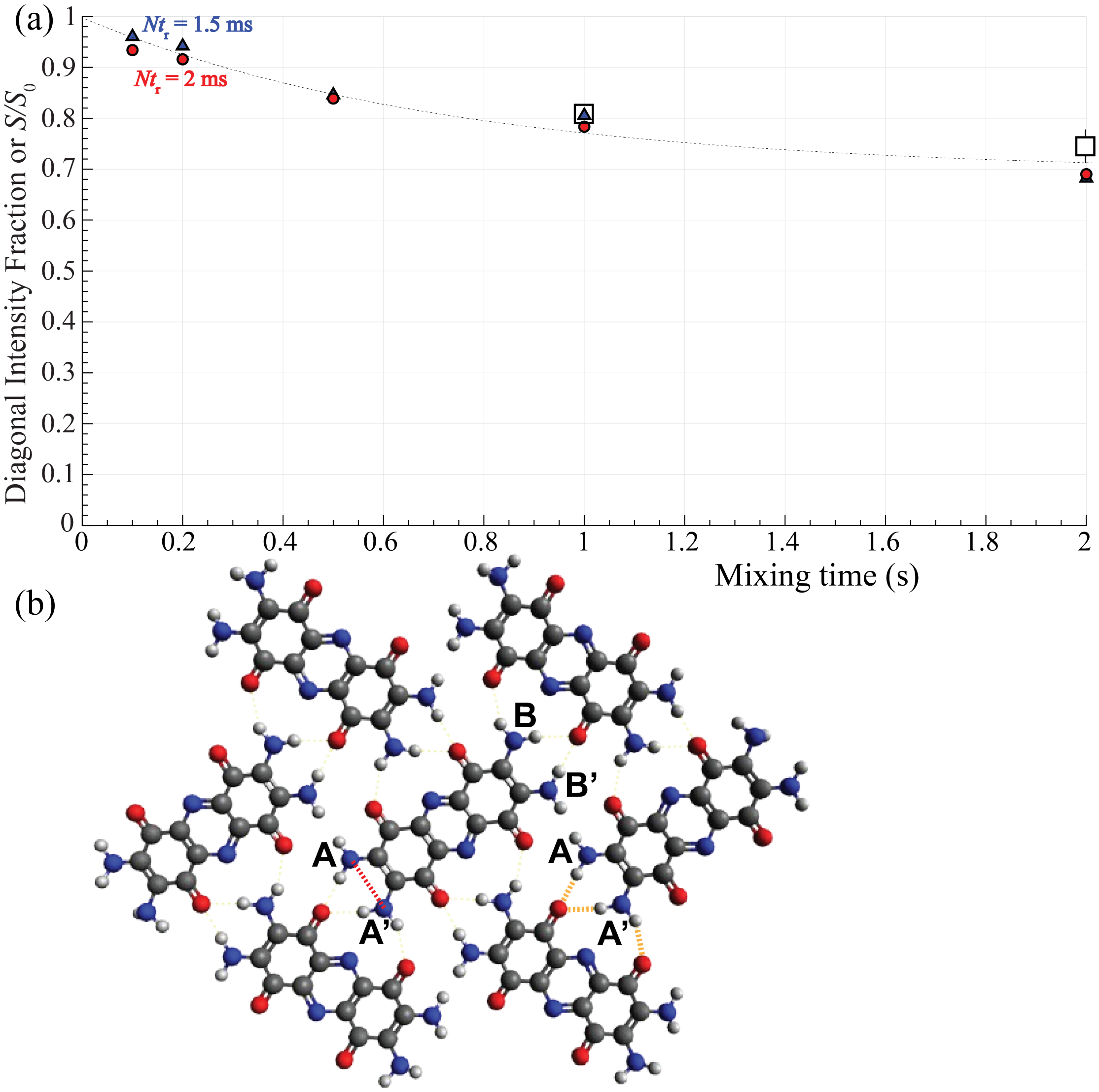

Figure S8. (a) ^{15}N exchange NMR in $^{15}N_6$-“TAQ” at 4 kHz MAS: ^{15}N CODEX S/S_0 of the high 69-ppm peak as a function of mixing time, for Nt_r = 1.5 ms (triangles) and 2 ms (circles), compared with (open squares) the average diagonal-peak fraction in 2D exchange ^{15}N NMR experiments also at 4 kHz MAS. The dashed curve is a guide to the eye. (b) Crystal structure of TAPT, with a short 2.8-Å intramolecular *ortho* N-N distance (dashed red line), while the shortest intermolecular N-N distances are 3.7 Å and 4.6 Å. A hypothetical model with *ortho* NH_2 groups that are chemically equivalent (A and A’; B and B’) and therefore would not produce intramolecular cross peaks is represented schematically. A and A’ would still be magnetically inequivalent and therefore would produce fast CODEX decay, with a rate at least twice faster than the decrease in the diagonal peak in a 2D exchange experiment, which would only detect slower intermolecular exchange. This is not observed in the experimental data in a), so we conclude that A and A’ have different isotropic chemical shifts. CODEX is also affected by slow motions of the amine group, which can explain the *slightly* faster CODEX decay in a).
In response to a request by a reviewer, we have also highlighted the three hydrogen bonds of the A and A’ amines in the right-most molecule (dashed orange lines). The corresponding N…O distances from top to bottom are 2.89 Å, 3.00 Å, and 3.07 Å.

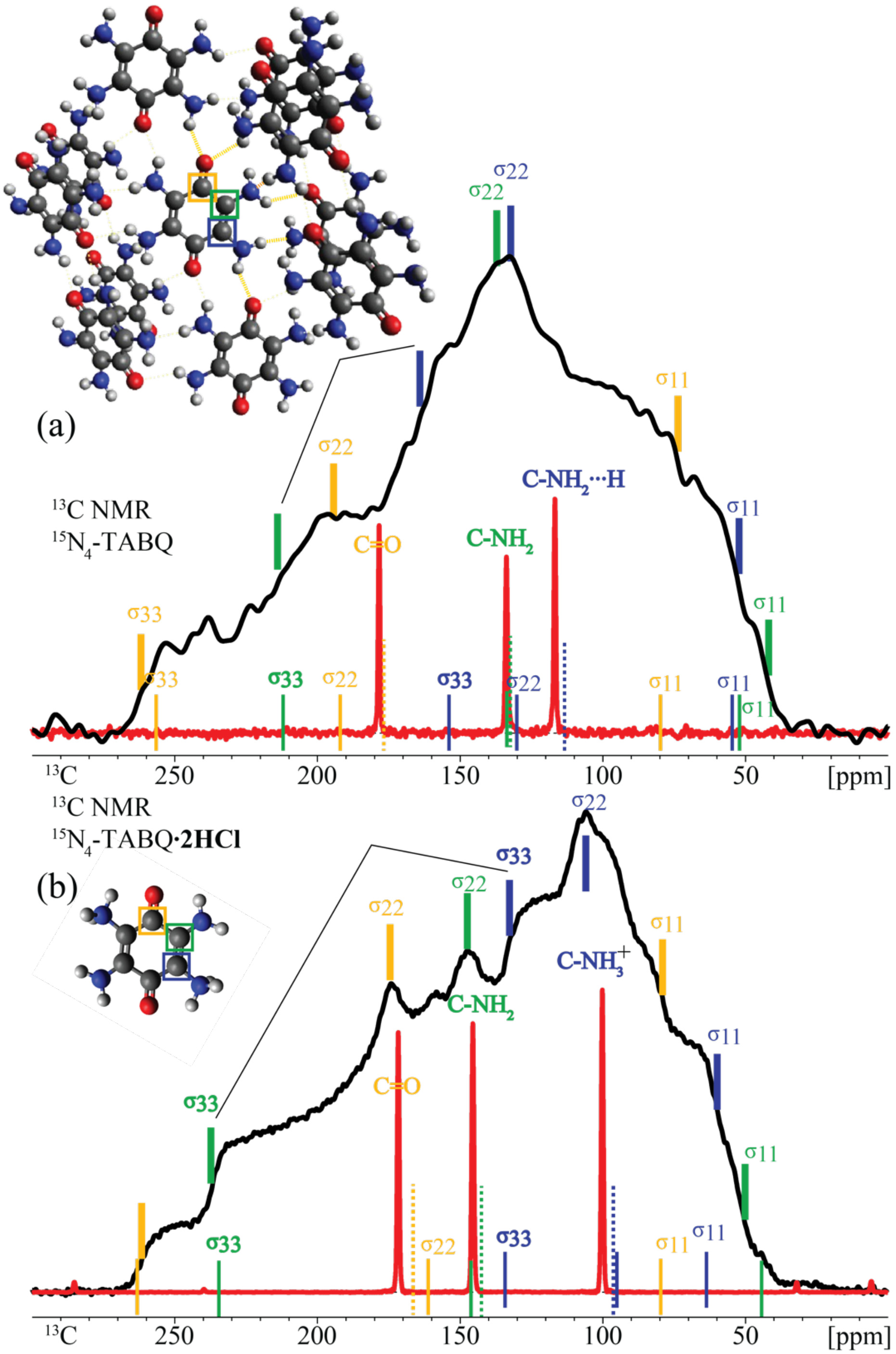

Figure S9. MAS ^{13}C NMR spectra (in red) and static ^{13}C spectra (CSA powder patterns) of (a) TABQ and (b) TABQ·2HCl precipitated from 6 M HCl. Long vertical dashed lines: quantum-chemically predicted isotropic chemical shifts; solid vertical lines on the x-axis – predicted principal values; above the static spectrum: corresponding principal values read off from inflection points and local maxima. Converging thin black lines at the top mark the change in the left-most (σ_{33}) principal value when the NH_2 group has accepted a proton. In response to a reviewer request, we have also highlighted the hydrogen bonds in the crystal structure in a). The corresponding N…N distance is 3.06 Å while the N…O distances are 2.96 and 3.1 Å.

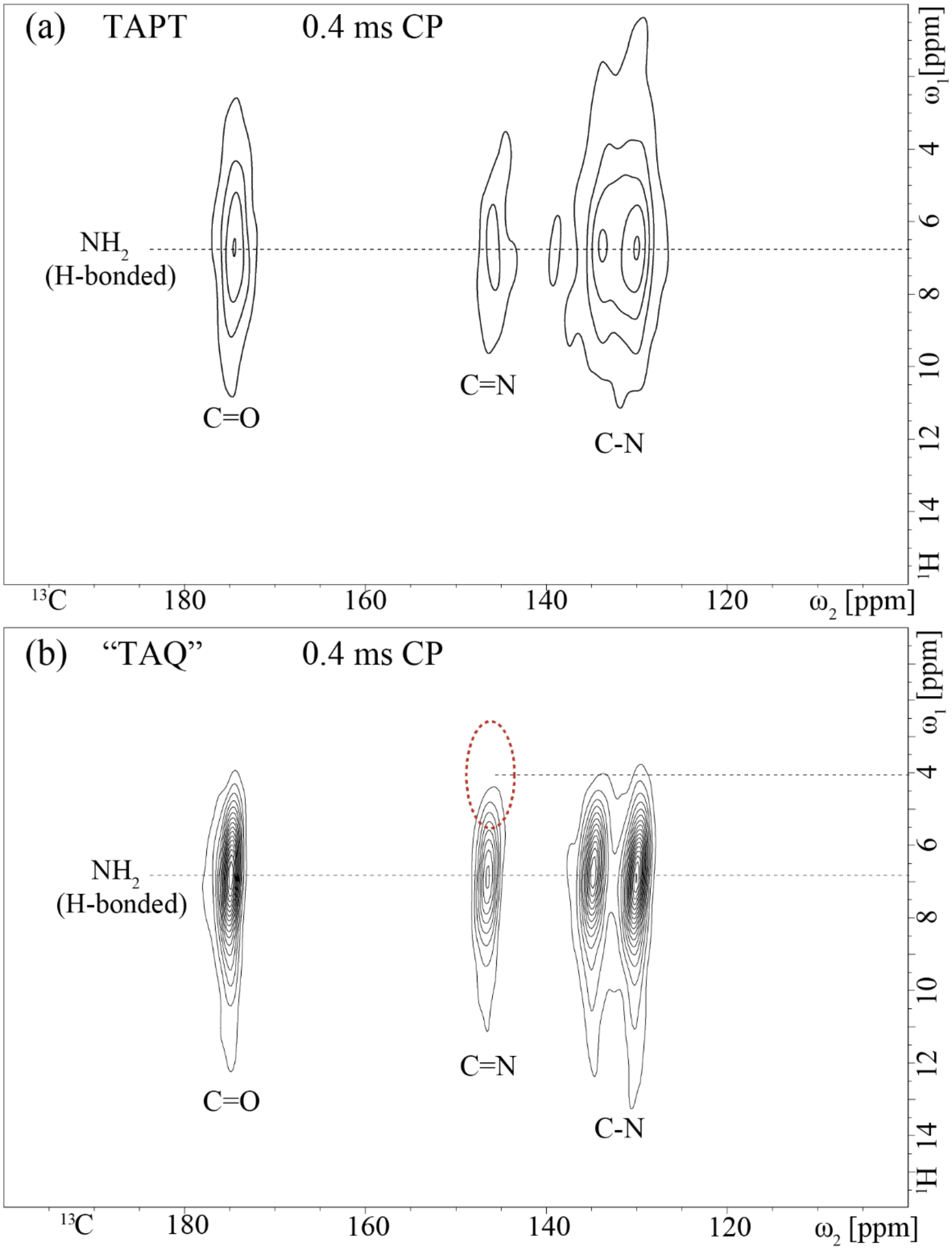

Figure S10. ^{1}H-^{13}C HetCor spectra of (a) TABQ and (b) "TAQ", consistently showing a single ^{1}H NMR peak near 6.7 ppm.

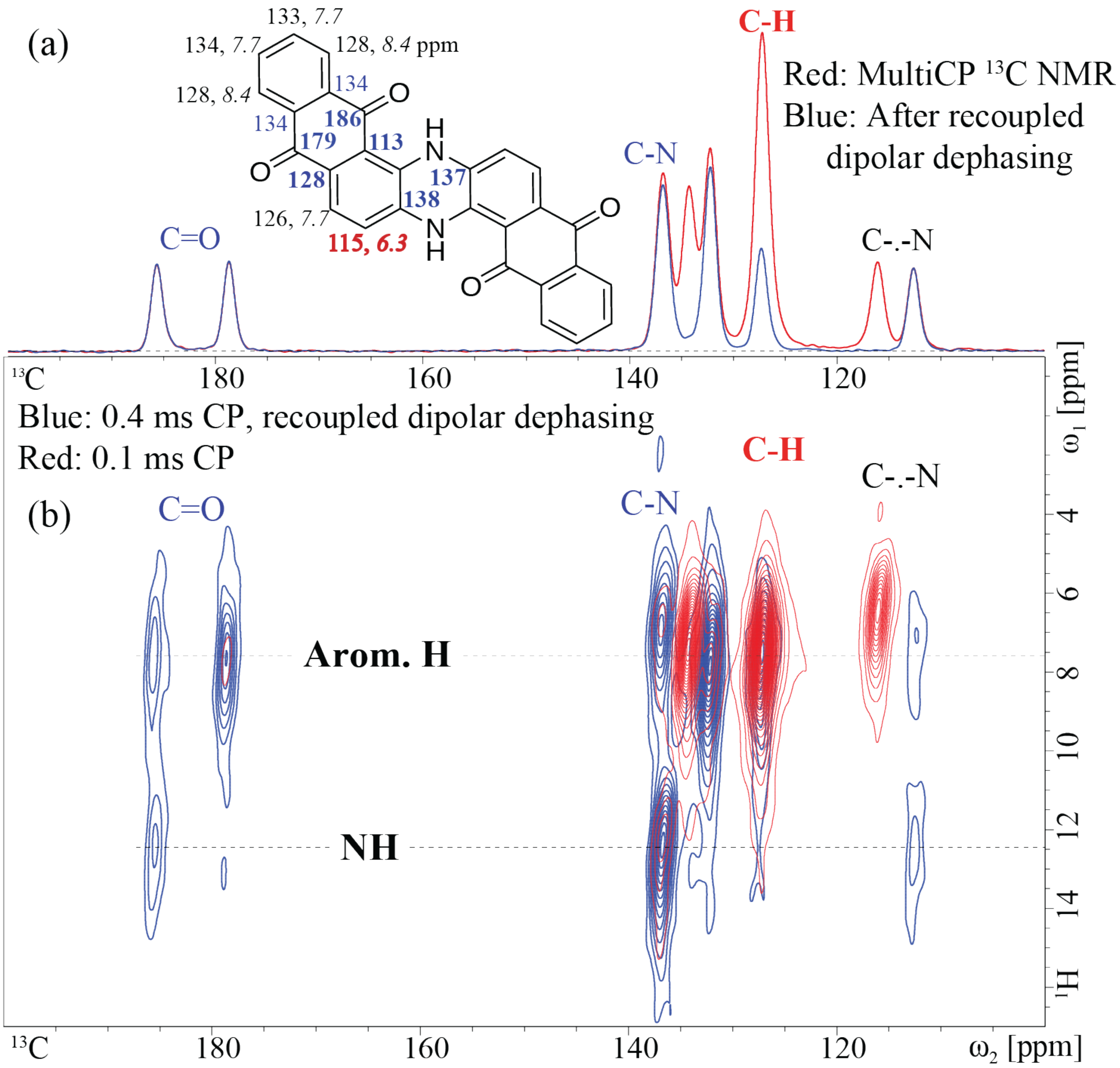

Figure S11. (a) MultiCP ^{13}C NMR and (b) ^{1}H-^{13}C HetCor spectra of indanthrone without (red) and with (blue) recoupled dipolar dephasing. Predicted ^{13}C and ^{1}H chemical shifts are shown next to the molecular structure in a).

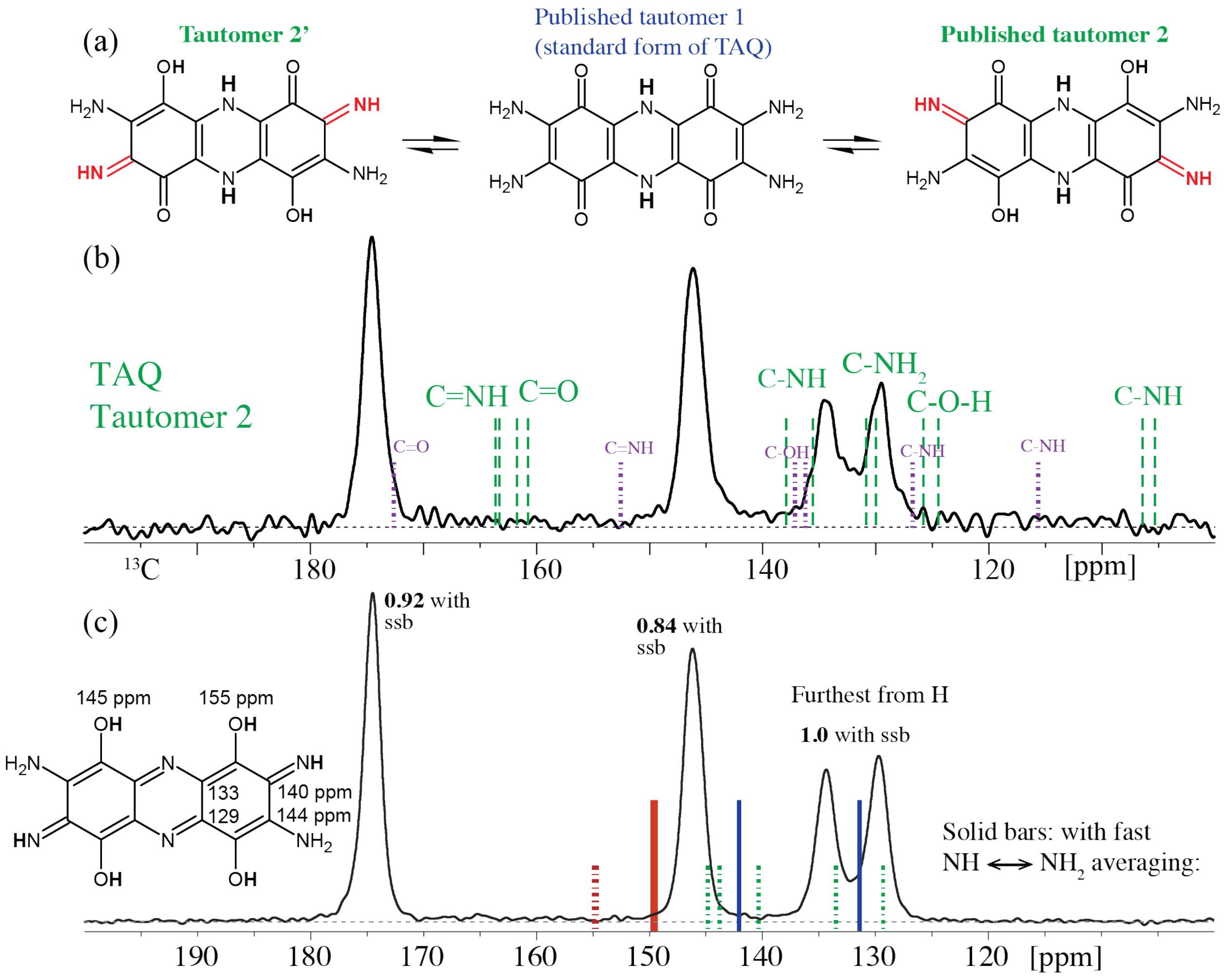

Figure S12. ^{13}C chemical shift predictions for tautomers of TAQ. (a) Tautomers proposed in the literature.[2] (b) Spectrum of "TAQ" with predicted chemical shifts of tautomer 2 from quantum-chemical calculations marked by dashed lines, and those from ACD/NMR by dash-dotted lines. (c) Tautomer of TAQ without hydrogen atoms bonded to the central ring, with predictions from ACD/NMR marked by dash-dotted lines; assignments are indicated in the structure on the left. In all cases, predicted chemical shifts are on average too low to match the shown experimental multiCP spectrum of "TAQ".

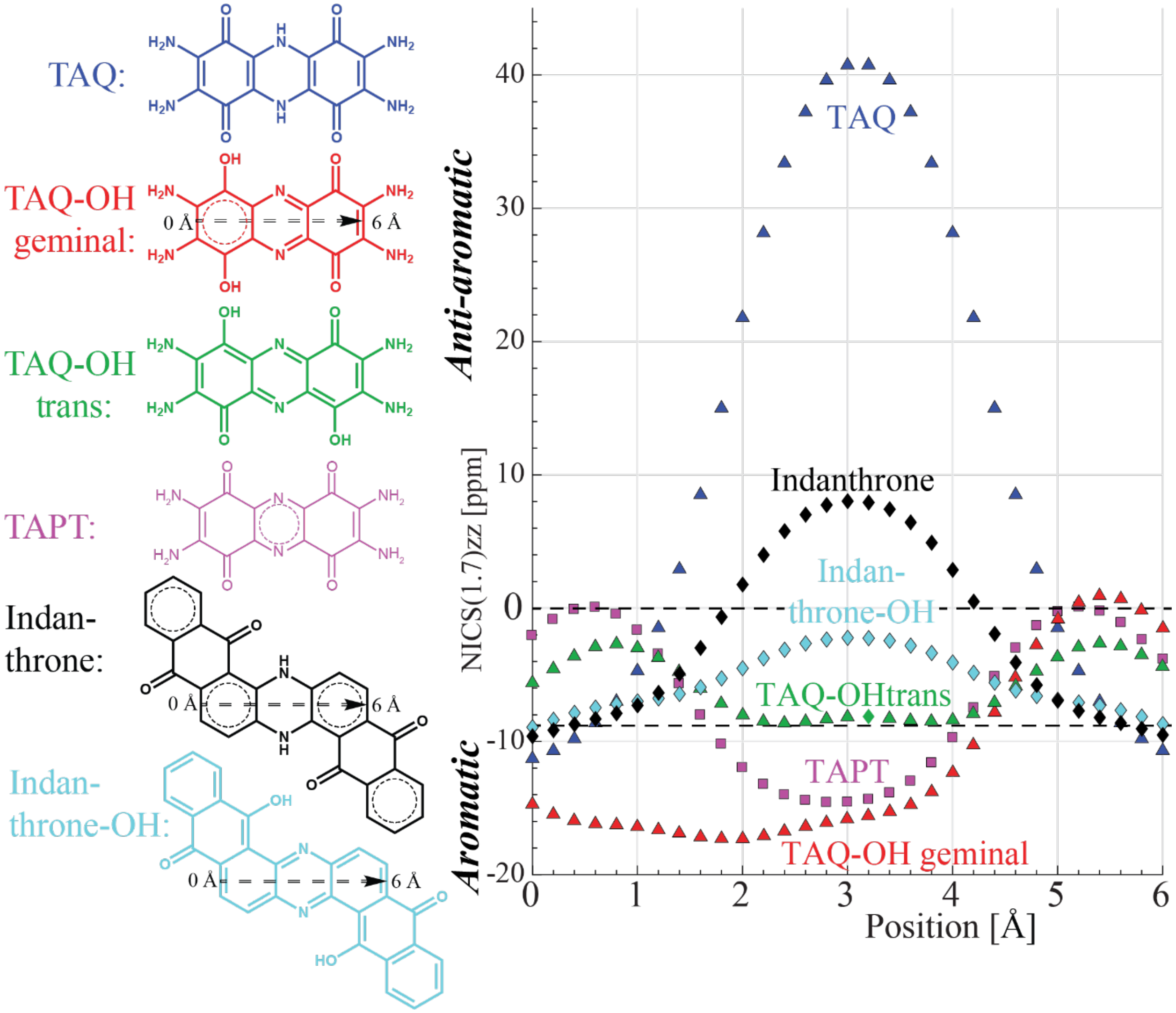

Figure S13. NICS aromaticity scans of TAQ, TAPT (see Figure S1), two TAQ tautomers that are reduced forms of TAPT, and indanthrone. Significantly negative values indicate aromatic rings, positive values anti-aromatic rings. All mobile sextets of electrons in the structures (indicated by dashed circles) are aromatic in the scans.

References Cited in the SI

(1) Li, Z.; Jia, Q.; Chen, Y.; Fan, K.; Zhang, C.; Zhang, G.; Xu, M.; Mao, M.; Ma, J.; Hu, W. A Small Molecular Symmetric All-organic Lithium-ion Battery. *Angew. Chem., Int. Ed.* **2022**, *61*, e202207221. DOI: 10.1002/anie.202207221.

(2) Chen, T.; Banda, H.; Wang, J.; Oppenheim, J. J.; Franceschi, A.; Dincă, M. A Layered Organic Cathode for High-energy, Fast-charging, and Long-Lasting Li-ion Batteries. *ACS Cent. Sci.* **2024**, *10*, 569-578. DOI: 10.1021/acscentsci.3c01478.

(3) Qiu, Q.; Sun, Z.; Joubran, D.; Li, X.; Wan, J.; Schmidt-Rohr, K.; Han, G. G. Optically Controlled Recovery and Recycling of Homogeneous Organocatalysts Enabled by Photoswitches. *Angew. Chem. Int. Ed.* **2023**, *62*, e202300723. DOI: 10.1002/anie.202300723.

(4) Bodenhausen, G.; Freeman, R.; Turner, D. L. Suppression of Artifacts in Two-dimensional J Spectroscopy. *J. Magn. Reson.* **1977**, *27*, 511-514. DOI: 10.1016/0022-2364(77)90016-6.

(5) Duan, P.; Schmidt-Rohr, K. Composite-pulse and Partially Dipolar Dephased multiCP for Improved Quantitative Solid-state ^{13}C NMR. *J. Magn. Reson.* **2017**, *285*, 68-78. DOI: 10.1016/j.jmr.2017.10.010.

(6) deAzevedo, E. R.; Hu, W.-G.; Bonagamba, T. J.; Schmidt-Rohr, K. Principles of Centerband-only Detection of Exchange in Solid-state Nuclear Magnetic Resonance, and Extension to Four-time Centerband-only Detection of Exchange. *J. Chem. Phys.* **2000**, *112*, 8988-9001. DOI: 10.1063/1.481511.

(7) Dixon, W.; Schaefer, J.; Sefcik, M.; Stejskal, E.; McKay, R. Total Suppression of Sidebands in CPMAS C-13 NMR. *J. Magn. Reson.* **1982**, *49*, 341-345. DOI: 10.1016/0022-2364(82)90199-8.

(8) Blumberg, W. E. Nuclear Spin-Lattice Relaxation Caused by Paramagnetic Impurities. *Phys. Rev.* **1960**, *119*, 79-84. DOI: 10.1103/PhysRev.119.79.

(9) Furman, G. B.; Kunoff, E. M.; Goren, S. D.; Pasquier, V.; Tinet, D. Nuclear spin-lattice relaxation via paramagnetic impurities in solids with arbitrary space dimension. *Phys. Rev. B* **1995**, *52*, 10182-10187. DOI: 10.1103/PhysRevB.52.10182.
